# A mechanovascular framework for pre-neoplastic microenvironmental dysregulation and early carcinogenesis

**DOI:** 10.3389/fonc.2026.1848102

**Published:** 2026-06-29

**Authors:** Amal Bhanu Vayakkattil, Aiswarya Sivan Pazhanchery, Varsha Vijayarajan, Udayabhanu Vayakkattil

**Affiliations:** 1 Department of Medicine, Al Ameen Hospital Kunnamkulam, Thrissur, Thrissur, India; 2 Department of Medicine, Sapthagiri Institute of Medical Sciences and Research Center, Bengaluru, India; 3 Department of Biochemistry, Gamma High Tech Laboratory, Ollur, Thrissur, India

**Keywords:** aerobic glycolysis, electrostatic metabolite partitioning, functional hypoxia, glycocalyx disruption, interstitial fluid pressure, mechanovascular framework, pre-neoplastic microenvironment

## Abstract

Aerobic glycolysis is a defining feature of many solid tumors; however, the upstream physiological conditions that initiate and stabilize this metabolic phenotype during early carcinogenesis remain incompletely understood. Here, we propose a mechanovascular framework in which chronic vasomotor dysregulation, endothelial glycocalyx disruption, low-grade inflammation, endothelial hyperpermeability, and impaired lymphatic drainage collectively contribute to elevated interstitial fluid pressure and progressive extracellular matrix remodeling prior to clinically detectable tumor formation. In this context, an endothelin-1-dominant vasomotor imbalance is suggested to increase capillary hydrostatic pressure and promote interstitial fluid accumulation. Erythrocyte mechanotransduction and shear-dependent adenosine triphosphate-nitric oxide signaling are considered integral to microvascular homeostasis, and their disruption may contribute to perfusion heterogeneity and impaired vascular regulation. These biomechanical alterations are associated with the activation of mechanosensitive signaling pathways that enhance glucose uptake and glycolytic flux while constraining mitochondrial pyruvate oxidation, thereby favoring a sustained glycolytic phenotype and cellular proliferation. Progressive matrix expansion increases the fixed negative charge density and may impose electrostatic constraints on solute mobility, contributing to spatial heterogeneity in metabolite distribution. Elevated extracellular lactate levels under these conditions may impair the metabolic fitness of immune cells and reduce their cytotoxic function. We further propose that functional hypoxia may arise from a transport-limited spatial dysregulation of oxygen delivery rather than solely from vascular insufficiency. At the system level, sustained microenvironmental stress is suggested to induce metabolic plasticity, which may be stabilized through epigenetic remodeling and ultimately consolidated by genetic alterations. Collectively, this framework identifies interstitial biomechanical and transport dysregulation as potential upstream drivers of metabolic reprogramming and immune suppression and suggests that restoring vascular-interstitial homeostasis may provide a rational strategy for early cancer interception.

## Graphical Abstract

**Figure:**
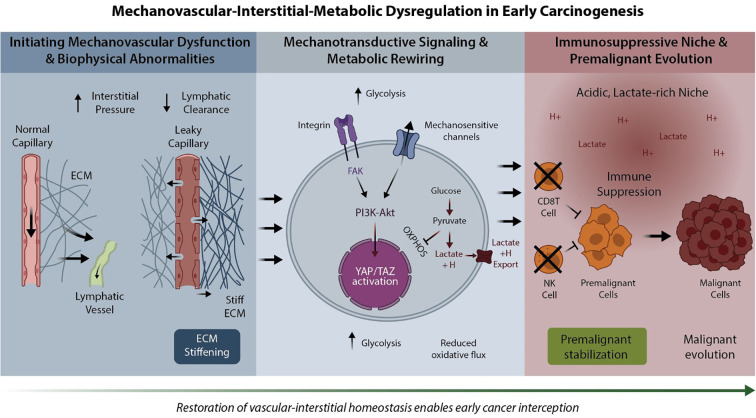

## 1 Introduction

Cancer remains a leading cause of morbidity and mortality worldwide, accounting for nearly 10 million deaths annually (1). Despite substantial advances in genomics, molecular pathology, and targeted therapeutics, the biological processes that initiate and sustain solid tumors at the tissue and microenvironmental levels remain incompletely understood. Although mutation-centric models explain many aspects of malignant behavior, they do not fully account for several consistent clinical observations, including tissue specificity of cancer incidence, the age-associated increase in risk, the presence of oncogenic mutations in histologically normal tissues, and variability in immune surveillance effectiveness. These observations suggest that carcinogenesis cannot be fully explained as a purely cell-autonomous genetic process but that it is also shaped by progressive alterations in the tissue microenvironment.

The importance of tissue context in cancer biology has long been recognized. Paget’s seed and soil hypothesis emphasizes the dependence of tumor growth on the properties of the host tissue (2, 3). While this concept has been extensively applied to metastatic progression, its relevance to primary tumor initiation remains less clearly defined. Notably, many solid tumors arise in tissues characterized by altered vascular tone, elevated interstitial fluid pressure, impaired lymphatic drainage, extracellular matrix remodeling, and metabolic reprogramming (4). Although these features are often interpreted as downstream consequences of tumor growth, accumulating evidence suggests that microenvironmental dysfunction, particularly in the setting of aging and chronic inflammatory, mechanical, and metabolic stress, may precede and influence early tumorigenic processes.

Under physiological conditions, tissue homeostasis is maintained through the coordinated regulation of capillary hydrostatic pressure, endothelial barrier integrity, plasma oncotic forces, and lymphatic drainage, ensuring efficient oxygen delivery, nutrient exchange, and metabolic waste removal (5, 6). With aging and prolonged exposure to stressors, this balance may progressively deteriorate. Persistent vasomotor dysregulation may increase capillary hydrostatic pressure and vascular permeability, promoting plasma extravasation, while lymphatic clearance and capillary reabsorption may become insufficient. As a result, interstitial fluid may accumulate and interstitial pressure may rise, reflecting a shift toward impaired microvascular and interstitial transport balance.

Elevated interstitial pressure imposes sustained mechanical loading on the extracellular matrix and resident cells, potentially increasing matrix stiffness and altering cellular signaling. Stromal and parenchymal cells respond to these biophysical changes through integrin-mediated adhesion complexes and mechanosensitive ion channels, including Piezo1 and transient receptor potential channels. Downstream signaling through focal adhesion kinase and Src family kinases is associated with the activation of central regulatory pathways such as PI3K–Akt–mTOR and YAP/TAZ, which support anabolic metabolism and cell survival under sustained mechanical stress (7, 8). This persistent mechanotransductive signaling may reduce dependence on transient growth factor inputs and favor stable adaptive states.

Mechanotransductive signaling is closely linked to metabolic reprogramming. The sustained activation of PI3K–Akt–mTOR and YAP/TAZ pathways is associated with increased glucose uptake and glycolytic flux, promoting lactate production even under normoxic conditions (9). In parallel, impairment of interstitial convection and lymphatic drainage may limit efficient metabolite clearance, favoring extracellular lactate accumulation within the tissue microenvironment. Accumulated lactate may further influence endothelial permeability, vascular tone, and matrix hydration, potentially reinforcing a feed-forward cycle of mechanical and metabolic dysregulation. In mechanically stressed microvascular environments, erythrocyte metabolism and transcapillary exchange may also contribute to local metabolite flux, although the extent of this contribution likely depends on local transport conditions (10).

The resulting lactate-enriched extracellular environment has important implications for immune surveillance. Cytotoxic T lymphocytes and natural killer cells depend on sustained glycolytic metabolism to support clonal expansion and effector function. Elevated extracellular lactate may reduce the transmembrane gradient required for lactate export, leading to intracellular accumulation, inhibition of glycolytic enzymes, and progressive metabolic exhaustion of immune effector cells (11). As immune cells traverse the interstitium and undergo lymphatic egress, exposure to such conditions may impair metabolic fitness and cytotoxic capacity, potentially contributing to localized immune tolerance prior to clinically detectable tumor formation.

These processes together define a premalignant microenvironment characterized by impaired perfusion, altered extracellular matrix mechanics, reduced interstitial transport, and spatially constrained metabolite clearance. Recognizing that such conditions may arise prior to overt genetic transformation supports the view of carcinogenesis as an evolutionary process shaped by microenvironmental selection pressures rather than being driven solely by mutation. In this context, therapeutic strategies aimed at restoring vascular regulation, normalizing interstitial transport, preserving matrix compliance, and improving metabolic clearance may reduce the probability that sporadic oncogenic events progress into clinically significant malignancies.

## 2 Glycolytic flux in cellular proliferation

Cell proliferation requires metabolic programs that simultaneously generate ATP and provide biosynthetic precursors for nucleotides, amino acids, and lipids. To meet these demands, proliferating cells preferentially adopt aerobic glycolysis, commonly referred to as the Warburg effect, even in the presence of oxygen. Although glycolysis yields less ATP per molecule of glucose than oxidative phosphorylation, its high catalytic throughput enables rapid ATP generation under conditions of sustained substrate availability, thereby supporting the elevated metabolic flux required for cellular growth (12). Continuous regeneration of cytosolic NAD+ by lactate dehydrogenase maintains redox balance and prevents glycolytic arrest, provided that lactate is efficiently exported via proton-coupled monocarboxylate transporters, thereby preserving a favorable NAD+/NADH ratio (13). It is important to note that this glycolytic emphasis is specific to the proliferative and pre-neoplastic context addressed by this framework; established cancers exhibit substantial metabolic plasticity, including transitions to oxidative phosphorylation, glutamine dependence, and fatty acid oxidation, as discussed in Section 4.2.

At the systems level, sustained glycolytic flux is governed by the coordinated interplay between substrate availability, enzymatic capacity, and efficient removal of metabolic end products. These factors establish glycolysis as a high-flux pathway operating under nonequilibrium steady-state conditions, in which reaction directionality is maintained by continuous substrate influx and product clearance rather than proximity to thermodynamic equilibrium. Flux control is distributed across key regulatory nodes, and growth-promoting signaling pathways further modulate throughput. In parallel, elevated extracellular glucose may suppress mitochondrial respiration through the Crabtree effect, reinforcing glycolytic dependence under proliferative conditions (14).

A defining feature of glycolysis in proliferating cells is the metabolic plasticity of its intermediates. Glucose-6-phosphate supports the pentose phosphate pathway for nucleotide and NADPH synthesis; 3-phosphoglycerate fuels serine synthesis, with serine further supporting folate-mediated one-carbon transfer required for nucleotide biosynthesis and methylation reactions; and dihydroxyacetone phosphate feeds lipid biosynthesis (15). The serine–glycine–one-carbon axis also intersects with glutamine-dependent anaplerotic carbon entry into the tricarboxylic acid cycle, providing a metabolic flexibility axis that becomes particularly relevant under conditions of constrained glycolytic flux. Through these branching pathways, glycolysis functions as an integrated bioenergetic and biosynthetic network that interfaces with amino acid and nucleotide metabolism to support biomass accumulation.

Proliferating cells operate under stringent temporal constraints, requiring rapid biomass expansion within a defined cell cycle interval. High glycolytic throughput enables simultaneous ATP production and precursor generation, minimizing the time required to reach this anabolic threshold. In contrast, exclusive reliance on oxidative phosphorylation, despite its higher ATP yield per glucose molecule, is constrained by mitochondrial capacity and is less adaptable to rapid fluctuations in biosynthetic demand. Within the mechanovascular framework proposed here, increased capillary filtration and endothelial permeability may enhance interstitial glucose availability, while mechanically induced PI3K–Akt signaling promotes glucose transporter expression, creating conditions that support sustained glycolytic flux (16, 17).

Growth-promoting signaling pathways further stabilize this metabolic configuration through convergent biochemical and mechanical inputs. Extracellular matrix stiffening, integrin clustering, cytoskeletal tension, and shear-dependent deformation activate PI3K–Akt–mTOR signaling axes, which collectively enhance glucose uptake and glycolytic capacity. These adaptations support progression through the G1-to-S phase transition and sustained anabolic growth (16, 17). While transient activation of glycolysis occurs in physiological contexts such as development, tissue repair, and immune activation, persistent activation is associated with pathological proliferation and represents a defining feature of oncogenesis.

In mechanically stressed tissues, stromal cells may exhibit elevated glycolytic flux and increased lactate export, contributing to extracellular lactate accumulation. Because activated immune effector cells, including CD8+ T cells and natural killer cells, depend on glycolysis for proliferation and cytokine production, elevated extracellular lactate and associated acidification may impair their function by reducing lactate efflux and disrupting metabolic reprogramming (11, 18). This interplay between stromal metabolism, tissue mechanics, and immune cell function highlights a systems-level linkage between metabolic flux and immune regulation within the pre-neoplastic tissue microenvironment.

### 2.1 Determinants of glycolytic flux: glucose uptake and lactate efflux

High glycolytic activity in proliferating cells depends on sustained glucose availability together with efficient clearance of metabolic end products. Diversion of glycolytic intermediates into anabolic pathways establishes a biosynthetic state that supports biomass accumulation and cell cycle progression, thereby tightly coupling glycolysis to proliferation (19, 20).

In proliferating cells, glycolysis is maintained as a sustained high-throughput pathway supporting both ATP generation and the continuous production of biosynthetic intermediates. This configuration persists despite dynamic regulation at key enzymatic nodes, enabling the maintenance of elevated pathway flux under both oxygenated and hypoxic conditions (19, 21). It should be noted that glycolytic dominance is not uniform across all cell cycle phases; G1-to-S phase transitions rely most heavily on glycolysis and anabolic intermediate generation, while oxidative metabolism may contribute more substantially during G2 and M phases, reflecting the dynamic and phase-dependent nature of proliferative bioenergetics.

Glucose uptake is mediated primarily by facilitative transporters such as GLUT1 and is regulated by growth factor signaling, concentration gradients, and mechanical cues (22). Activation of PI3K–Akt signaling increases GLUT1 expression and promotes its localization to the plasma membrane, maintaining intracellular glucose levels sufficient to sustain glycolysis and biosynthesis (23). Because both glucose and lactate transport occur via facilitated diffusion, sustained glycolytic flux depends on preservation of favorable concentration and electrochemical gradients across the plasma membrane rather than on active transport mechanisms.

Within the glycolytic pathway, phosphofructokinase-1 functions as a central metabolic integrator, inhibited by ATP, citrate, and acidification and activated by fructose-2,6-bisphosphate (24, 25). Pyruvate kinase M2 provides a complementary regulatory node balancing ATP production with anabolic demand. Its activity is enhanced by fructose-1,6-bisphosphate, serine, and SAICAR and reduced by ATP, pyruvate, alanine, phenylalanine, and post-translational modifications (26). These regulatory mechanisms together enable the redistribution of carbon flux toward biosynthetic pathways without suppressing the overall glycolytic throughput.

Reduced pyruvate kinase M2 activity promotes the accumulation of upstream intermediates, facilitating their diversion into anabolic pathways. Similarly, moderated phosphofructokinase-1 activity allows the accumulation of glucose-6-phosphate, supporting entry into the pentose phosphate pathway. The oxidative branch generates NADPH for reductive biosynthesis and redox homeostasis, while the nonoxidative branch supplies ribose-5-phosphate and returns intermediates to glycolysis under conditions of sustained substrate influx (27). In cancer cells, this regulatory architecture is further reinforced by transcriptional and post-transcriptional mechanisms, including HIF-1 alpha activation, EGFR-NF-kB signaling, and c-Myc-dependent alternative splicing (28–31).

Sustained glycolysis also reinforces its own persistence through mitochondrial gating, as elaborated in Section 2.3. Elevated intracellular ATP, NADH, and acetyl-CoA activate pyruvate dehydrogenase kinase, which phosphorylates and inhibits the pyruvate dehydrogenase complex, limiting mitochondrial pyruvate oxidation and directing flux toward lactate production (32, 33). This feedforward regulation contributes to stabilization of the glycolytic phenotype.

Lactate export is mediated by proton-coupled monocarboxylate transporters, primarily MCT1 and MCT4, whose differential kinetic roles under conditions of elevated extracellular lactate and impaired clearance are examined in detail in Section 2.8 (34). The efficiency of lactate export depends on transmembrane gradients of both lactate and protons and therefore may be reduced under conditions of elevated extracellular lactate or impaired interstitial clearance. Mechanotransductive signaling through YAP and TAZ further modulates the expression of glycolytic and transport-related genes in mechanically stressed tissues, supporting metabolic adaptation to physical cues (35).

The co-export of lactate and protons acidifies the pericellular environment, influencing stromal, endothelial, and immune cell behavior. The consequences of this efflux constraint for immune effector cell metabolism are elaborated in Section 3.

In sum, the concerted regulation of glucose influx, glycolytic intermediate redistribution, and lactate export, together with the preservation of favorable transmembrane and interstitial gradients, enables proliferating cells to maintain high glycolytic throughput while coupling ATP generation with continuous anabolic expansion.

### 2.2 Intrinsic constraints on glycolytic flux

While transporter capacity and enzyme abundance define the theoretical upper limit of glycolytic throughput, flux through the pathway is continuously modulated by intrinsic feedback mechanisms that align metabolic activity with cellular energetic and biosynthetic demand. Glycolysis therefore operates as a dynamically regulated network in which flux distribution prevents intermediate overaccumulation while enabling coordinated anabolic integration.

A primary layer of regulation arises from product-mediated feedback. Accumulation of glucose-6-phosphate allosterically inhibits hexokinase, coupling glucose phosphorylation to downstream metabolic capacity (36). Because several upstream reactions operate near thermodynamic equilibrium, intermediate accumulation propagates backward through the pathway, imposing resistance to further flux when downstream utilization is limited. Conversely, consumption of intermediates by branching pathways lowers local metabolite concentrations, shifts the mass–action ratio, and facilitates forward flux. This bidirectional responsiveness depends on intermediate concentrations exceeding the kinetic thresholds of downstream enzymes.

This threshold dependence establishes a gating mechanism for biosynthetic branching. Under basal conditions, branch-initiating enzymes, including glucose-6-phosphate dehydrogenase (pentose phosphate pathway), phosphoglycerate dehydrogenase (serine synthesis), and glycerol-3-phosphate acyltransferase (lipid biosynthesis), operate at metabolically negligible rates, and their pathways remain functionally quiescent. Importantly, this quiescence does not arise from substrate concentrations falling below enzyme Km values. It rather reflects the relatively low maximal catalytic capacity of these enzymes compared with the high-capacity mainstream glycolytic enzymes that process the same intermediates.

Glucose-6-phosphate dehydrogenase, for example, has a lower Km for glucose-6-phosphate than glucose-phosphate isomerase, indicating higher substrate affinity. However, its substantially lower Vmax limits its contribution to flux when glucose-phosphate isomerase operates near capacity. As a result, diversion into the pentose phosphate pathway remains minimal under basal conditions. In addition, glucose-6-phosphate dehydrogenase is subject to product inhibition by NADPH, further suppressing the activity when the NADPH/NADP+ ratio is high.

Branch pathway activation therefore requires the simultaneous fulfillment of two conditions. First, intermediate concentrations must rise beyond the processing capacity of mainstream glycolysis, generating substrate overflow toward low-Vmax branch enzymes. Second, product inhibition must be relieved through active consumption of branch products. As glycolytic influx increases under growth-promoting signals, both conditions are progressively satisfied, allowing branch pathways to achieve metabolically significant flux despite their intrinsically limited catalytic capacity.

This overflow dynamic is itself gated by two irreversible regulatory checkpoints within the mainstream glycolytic stream. Phosphofructokinase-1 and pyruvate kinase M2 constrain intermediate accumulation at low substrate influx, keeping concentrations below branch activation thresholds. As glucose uptake increases, these constraints are progressively relaxed, permitting upstream intermediates to accumulate and spill into biosynthetic pathways. Under conditions of maximal PI3K–Akt signaling and GLUT1 upregulation, this transition becomes abrupt, enabling the coordinated activation of multiple anabolic branches.

The regulatory configuration of PKM2 is particularly critical. In its low-activity dimeric state, PKM2 limits the conversion of phosphoenolpyruvate to pyruvate, preventing premature depletion of upstream intermediates and sustaining an expanded metabolite pool above biosynthetic activation thresholds. In this way, glycolysis is reconfigured from a predominantly ATP-generating pathway into a coordinated biosynthetic platform, providing a mechanistic basis for the Warburg phenotype as an anabolic adaptation rather than solely an energetic one.

Redox balance and intracellular pH impose an additional constraint on glycolytic flux. Glyceraldehyde-3-phosphate dehydrogenase directly couples pathway throughput to NAD+ availability. Lactate dehydrogenase, a high-capacity near-equilibrium enzyme, sustains NAD+ regeneration by driving the reduction of pyruvate to lactate, thereby maintaining the thermodynamic favorability of upstream reactions. Impaired lactate efflux diminishes this forward pull, leading to NAD+ limitation and reduced glycolytic throughput. Concurrent proton accumulation lowers intracellular pH and inhibits phosphofructokinase-1 activity (25, 37).

These regulatory layers therefore ensure that glycolytic throughput remains tightly coupled to the integrated demands of substrate supply, intrinsic enzymatic capacity, and product clearance efficiency. Under sustained nutrient supply and proliferative signaling, efficient lactate export preserves redox balance and supports high glycolytic throughput compatible with anabolic growth. In contrast, reduced transporter activity or diminished metabolic demand shifts the system toward a maintenance-oriented state of constrained flux. Within the mechanovascular framework proposed here, impaired interstitial lactate clearance amplifies these intrinsic regulatory constraints: by limiting lactate dehydrogenase-mediated NAD+ regeneration and disrupting redox balance, independent of intracellular enzymatic capacity, extracellular lactate accumulation imposes a microenvironmental restriction on glycolytic flux, establishing a direct mechanistic link between vascular dysfunction, metabolite clearance, and the emergence of glycolytic and redox dysregulation during early tumorigenesis.

### 2.3 PDH regulation and glycolytic flux in the Warburg effect

The pyruvate node represents a critical metabolic decision point at which carbon flux is partitioned between mitochondrial oxidation and lactate production. Regulation at this node determines whether glycolytic carbon is committed to oxidative metabolism or retained within the cytosolic redox cycle.

Under physiological conditions, rising intracellular ATP inhibits phosphofructokinase-1, shifts pyruvate kinase M2 toward lower activity states, and promotes the activation of pyruvate dehydrogenase kinase. This kinase phosphorylates and suppresses the pyruvate dehydrogenase complex (38). The pyruvate dehydrogenase complex functions as a tightly regulated and comparatively capacity-limited gatekeeper for mitochondrial carbon entry into the tricarboxylic acid cycle. Its activity is limited by ATP, NADH, and acetyl-CoA through pyruvate dehydrogenase kinase, ensuring that mitochondrial oxidation remains aligned with cellular energy demand (33).

In contrast, lactate dehydrogenase operates as a high-capacity near-equilibrium enzyme whose activity is governed primarily by substrate availability and cytosolic redox state. This enables rapid interconversion of pyruvate and lactate in response to changes in glycolytic flux (33).

As glycolytic throughput increases, this kinetic and regulatory asymmetry becomes functionally significant. Activity of the pyruvate dehydrogenase complex becomes progressively constrained by regulatory inhibition and intrinsic capacity limits, whereas lactate dehydrogenase continues to operate at high velocity. As a result, excess pyruvate is increasingly directed toward lactate production, particularly when lactate export remains efficient. Elevated ATP levels further reinforce this configuration by promoting pyruvate dehydrogenase kinase activity, thereby strengthening mitochondrial gating in relation to overall cellular energy status (33).

In proliferating and transformed cells, enhanced glucose uptake driven by increased GLUT1 expression maintains high intracellular substrate availability. At the same time, the increased expression of monocarboxylate transporters facilitates continuous lactate efflux, limiting product accumulation and preserving cytosolic redox balance (39, 40). Additional modulation occurs at phosphofructokinase-1 and pyruvate kinase M2. Fructose-2,6-bisphosphate strongly activates phosphofructokinase-1, while fructose-1,6-bisphosphate, serine, and mitogen-associated post-translational modifications favor higher pyruvate kinase M2 activity states, allowing glycolytic flux to remain elevated despite high intracellular ATP (24, 41).

**Figure 1:**
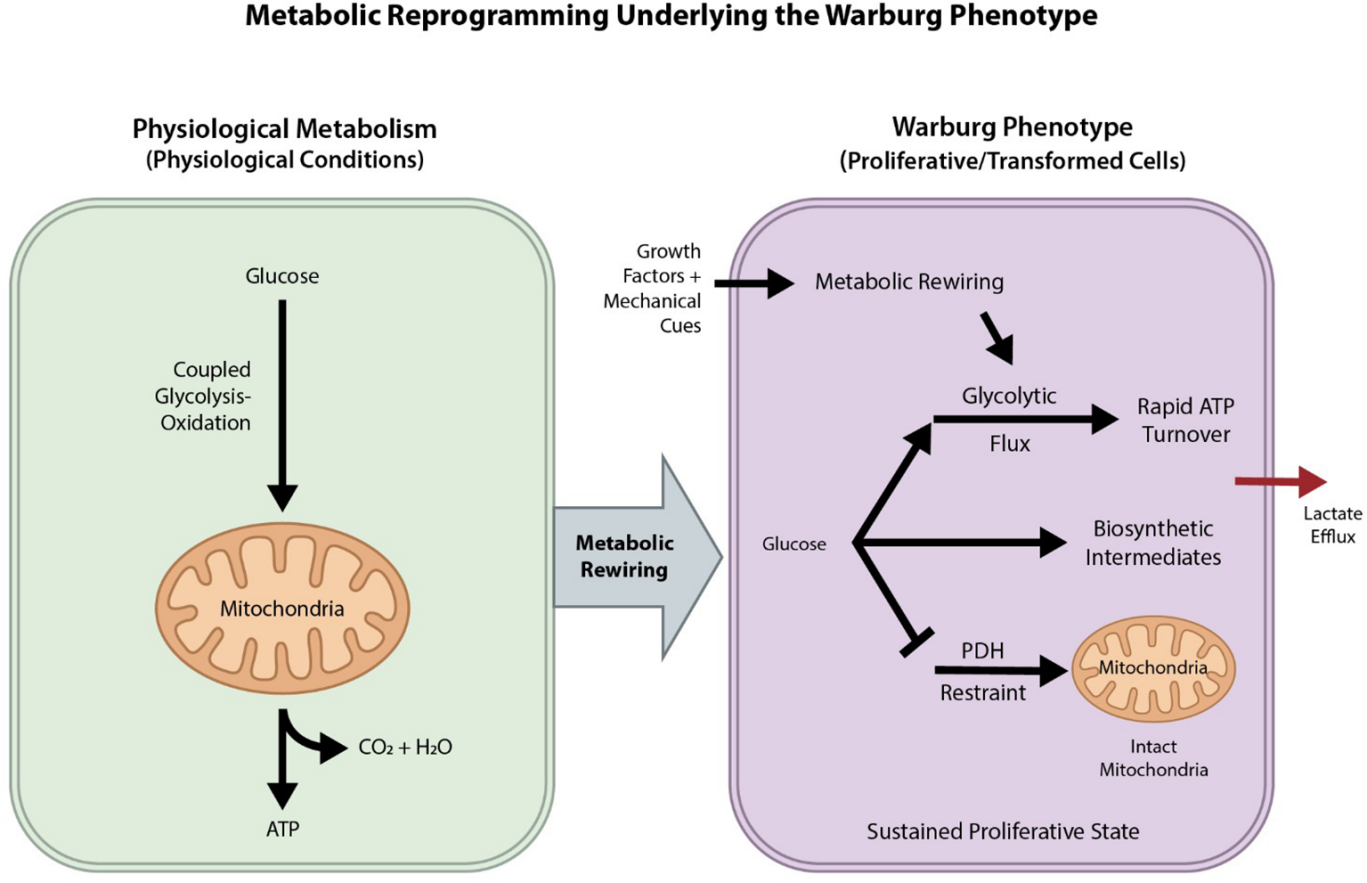
Metabolic reprogramming underlying the Warburg phenotype. The figure compares aerobic glucose metabolism under physiological conditions (left panel) with the Warburg phenotype characteristic of proliferating and transformed cells (right panel). Under normal conditions, glucose undergoes coupled glycolysis–oxidation, with pyruvate committed to mitochondrial oxidation, yielding CO_2_, H_2_O, and ATP. In the Warburg phenotype, exposure to growth factors and mechanical cues drives metabolic rewiring in which high glycolytic flux supports rapid ATP turnover per unit time and the continuous generation of biosynthetic intermediates. Pyruvate entry into the mitochondria is constrained by pyruvate dehydrogenase complex restraint (PDH restraint), redirecting carbon toward lactate production and efflux. Importantly, the mitochondria remain structurally intact, reflecting regulatory gating rather than organelle loss. This metabolic configuration supports the simultaneous bioenergetic and biosynthetic demands of sustained cellular proliferation. PDH, pyruvate dehydrogenase complex; OXPHOS, oxidative phosphorylation; ATP, adenosine triphosphate.

Under these integrated conditions, sustained suppression of the pyruvate dehydrogenase complex together with continued upstream glycolytic flux channels pyruvate toward lactate production. Continuous lactate export enables lactate dehydrogenase to maintain rapid turnover while preserving cytosolic redox balance. This coordinated configuration, characterized by increased glucose uptake, constrained mitochondrial pyruvate oxidation, and sustained lactate efflux, is characteristic of the metabolic phenotype of the Warburg effect (15) (**Figure 1**). It supports rapid ATP generation while preserving carbon skeletons for anabolic biosynthesis, thereby providing a robust metabolic foundation for cellular proliferation and oncogenic transformation.

### 2.4 PI3K–Akt signaling in the integration of mechanical cues and metabolic reprogramming

The PI3K–Akt pathway occupies a central position in this framework because it serves as the principal convergence node for multiple mechanosensory inputs—integrin–focal adhesion kinase signaling, Piezo1-mediated calcium influx, and glycocalyx deformation-dependent shear sensing—while simultaneously governing the metabolic outputs most relevant to pre-neoplastic reprogramming. Parallel mechanotransductive effectors including Rho-ROCK, MAPK-ERK, and NF-kappaB also contribute to the mechanovascular signaling cascade, providing mechanistic redundancy and context-dependent modulation, and are addressed in the sections that follow.

The PI3K–Akt pathway functions as a central integrative node that couples growth signals, metabolic programming, and mechanical inputs to coordinate nutrient uptake, anabolic biosynthesis, and cell survival through mTOR signaling (42). Under physiological conditions, this integration maintains alignment between metabolic output and cellular demand. However, when PI3K–Akt activation becomes sustained, cells may progressively reduce sensitivity to intrinsic nutrient sensing mechanisms and to growth-factor-dependent and contact-inhibition-mediated constraints that normally limit glucose uptake, glycolytic throughput, and proliferative commitment.

Pathway activation is initiated when ligands such as insulin or insulin-like growth factor 1 bind to receptor tyrosine kinases, leading to the recruitment of class I PI3K and conversion of phosphatidylinositol 4,5-bisphosphate to phosphatidylinositol 3,4,5-trisphosphate. The accumulation of phosphatidylinositol 3,4,5-trisphosphate recruits Akt to the plasma membrane via its pleckstrin homology domain, enabling co-localization with phosphoinositide-dependent kinase 1 for phosphorylation at Thr308 and subsequent phosphorylation by mTOR complex 2 at Ser473 for full activation (43). Activated Akt enhances glucose uptake by promoting glucose transporter expression and trafficking and by stimulating hexokinase activity, reinforcing the entry of glucose into glycolysis.

Downstream of Akt, mTOR complex 1 promotes anabolic metabolic reprogramming by inducing glycolytic enzyme expression, enhancing pentose phosphate pathway flux, and stimulating lipid and protein biosynthesis, mediated in part through hypoxia-inducible factor 1-alpha and c-Myc-dependent transcriptional programs (44, 45). Through these coordinated outputs, PI3K–Akt signaling supports a high-flux metabolic state compatible with biomass accumulation and cell cycle progression.

Mechanical cues constitute a major parallel regulatory layer converging on growth and survival pathways, including the PI3K–Akt axis. Integrins, focal adhesion complexes, and mechanosensitive ion channels sense extracellular matrix stiffness, shear stress, tensile strain, and related physical forces, converting mechanical inputs into biochemical signals that modulate PI3K–Akt activity (46, 47). Integrin engagement activates focal adhesion kinase and Src family kinases, promoting localized PI3K recruitment and phosphatidylinositol 3,4,5-trisphosphate accumulation at the plasma membrane (48). In parallel, mechanical loading activates mechanosensitive ion channels such as Piezo1, leading to calcium influx that interfaces with receptor-proximal signaling networks; bidirectional interactions between calcium and PI3K–Akt pathway components can amplify downstream proliferative and metabolic signaling (49, 50). These convergent inputs are together associated with enhanced PI3K–Akt signaling, increased mTOR activity, upregulation of glucose transporter expression, and metabolic reprogramming toward high glycolytic throughput (47).

Cytoskeletal tension and actin polymerization transmit mechanical forces to the nucleus, modulating Hippo pathway activity and facilitating the nuclear accumulation of YAP and TAZ (51, 52). Although the linker of nucleoskeleton and cytoskeleton complex mechanically couples cytoskeletal forces to nuclear architecture, YAP and TAZ activation is primarily governed by actin dynamics and Hippo pathway inhibition rather than direct nuclear deformation. In parallel, mechanotransduction activates myocardin-related transcription factor A and nuclear factor kappa B, coordinating transcriptional programs associated with proliferation, metabolic remodeling, and inflammatory signaling in mechanically stressed tissues (53).

Multiple regulatory mechanisms constrain PI3K–Akt pathway activity. The lipid phosphatase PTEN antagonizes PI3K signaling by dephosphorylating phosphatidylinositol 3,4,5-trisphosphate to phosphatidylinositol 4,5-bisphosphate, limiting Akt activation once upstream signals decline (54). Additional feedback mechanisms include receptor-proximal inhibition mediated by growth factor receptor-bound protein 10, suppression of insulin receptor substrates through mTOR complex 1 and S6 kinase signaling, and phosphatase-mediated attenuation of Akt activity (42). These layers together ensure that PI3K–Akt activation remains transient under physiological conditions.

When mechanical loading coincides with growth factor stimulation, PI3K recruitment and localized phosphoinositide signaling at the plasma membrane may be prolonged. This shifts the dynamic balance between phosphatidylinositol 3,4,5-trisphosphate generation and PTEN-mediated dephosphorylation, primarily reflecting sustained upstream receptor and adhesion-associated signaling rather than direct PTEN inhibition. Consequently, net phosphatidylinositol 3,4,5-trisphosphate accumulation is favored, with mechanical inputs acting as cooperative modulators that enhance receptor-proximal signaling efficiency, stabilize PI3K membrane association, and spatially confine phosphoinositide production.

Under physiological conditions, such signaling dominance is transient, as observed in development, wound repair, and adaptive tissue remodeling. In contrast, chronic upstream stimulation, including PTEN loss, activating PIK3CA mutations, Akt amplification, aberrant receptor tyrosine kinase activity, and persistent microenvironmental stress, is associated with sustained phosphatidylinositol 3,4,5-trisphosphate accumulation and prolonged Akt activation (55).

Sustained extracellular matrix stiffening, inflammation, and elevated interstitial pressure maintain focal adhesion kinase, Src, and Rho-associated kinase signaling, while repeated tissue deformation activates Piezo1 channels, generating recurrent calcium influx that may contribute to sustained PI3K–mTOR activity even in the absence of strong mitogenic stimulation (56). Persistent mechanical signaling may also indirectly modulate PTEN expression, stability, or subcellular localization through cytoskeletal remodeling and microRNA-mediated regulatory mechanisms rather than uniformly causing direct functional inhibition (57). As PTEN buffering capacity becomes insufficient relative to sustained phosphatidylinositol 3,4,5-trisphosphate production, PI3K–Akt activation may shift from a reversible adaptive response to a self-reinforcing pathological state (55).

In biological systems, prolonged dominance of a high-flux metabolic and signaling state may become epigenetically stabilized, particularly under conditions of sustained proliferative turnover. This stabilization reinforces glycolytic bias, anabolic signaling, and growth-permissive transcriptional programs governed by YAP, TAZ, myocardin-related transcription factor A, and nuclear factor kappa B, supporting early neoplastic expansion within mechanically dysregulated tissue microenvironments (9, 47).

Mechanochemical amplification of PI3K–Akt signaling may follow a hierarchical cellular pattern. Stromal cells, including fibroblasts, endothelial cells, and perivascular support cells, are exposed to sustained mechanical loading from elevated interstitial pressure and matrix stiffening and may therefore exhibit earlier and more persistent PI3K–Akt activation (57). Parenchymal cells become progressively engaged as tissue-level mechanical stress increases. Immune cells may experience comparatively lower sustained mechanical stress during routine interstitial migration due to their deformability and amoeboid motility, though they can encounter transient localized mechanical loading during diapedesis. These observations suggest that sustained PI3K–Akt signaling is driven primarily by tissue-level mechanical dysregulation rather than by continuous immune cell mechanostimulation.

**Figure 2:**
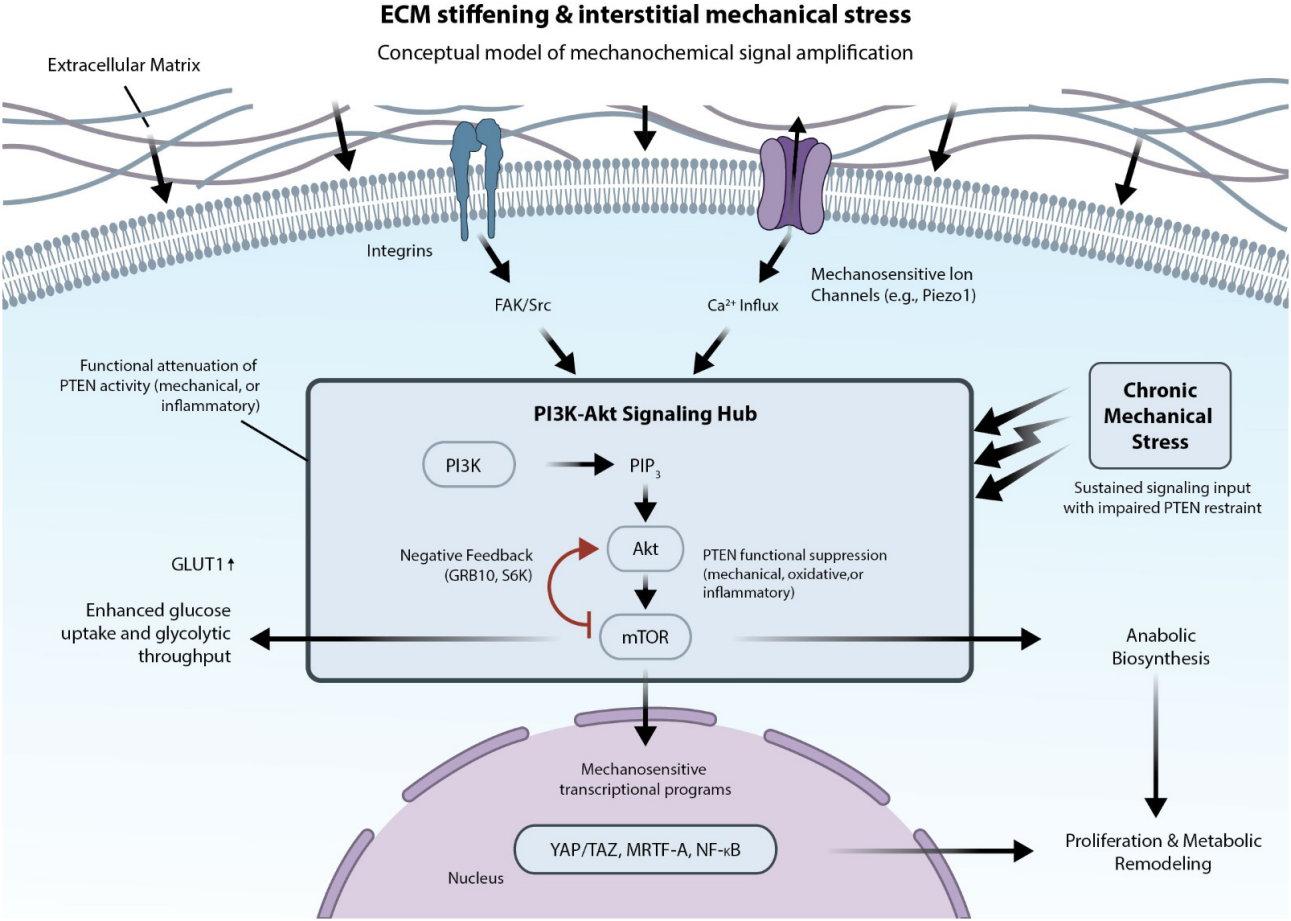
ECM stiffening and interstitial mechanical stress: A conceptual model of mechanochemical signal amplification through the PI3K–Akt signaling hub. The figure illustrates dual mechanosensing inputs that converge on the PI3K–Akt signaling hub under conditions of extracellular matrix (ECM) stiffening and sustained interstitial mechanical stress. Mechanical forces transmitted through the ECM activate two parallel receptor systems at the plasma membrane: integrins, which signal through focal adhesion kinase (FAK) and Src kinases, and mechanosensitive ion channels, including Piezo1, which mediate Ca^2+^ influx. Both inputs converge on the PI3K–Akt signaling hub, driving the conversion of phosphatidylinositol 4,5-bisphosphate (PIP2) to phosphatidylinositol 3,4,5-trisphosphate (PIP3) and subsequent Akt activation, which stimulates mTOR-dependent anabolic programs. Negative feedback via GRB10 and S6 kinase normally constrains this activation; however, under chronic mechanical stress, sustained signaling input may functionally attenuate PTEN activity through mechanical, oxidative, or inflammatory mechanisms, shifting the balance toward net PI3K–Akt activation. Downstream outputs include the upregulation of glucose transporter 1 (GLUT1) expression, enhanced glucose uptake and glycolytic throughput, anabolic biosynthesis, and activation of mechanosensitive transcriptional programs in the nucleus, including YAP/TAZ, MRTF-A, and NF-kB, promoting proliferation and metabolic remodeling. Akt, protein kinase B; ECM, extracellular matrix; FAK, focal adhesion kinase; GLUT1, glucose transporter 1; GRB10, growth factor receptor-bound protein 10; MRTF-A, myocardin-related transcription factor A; mTOR, mechanistic target of rapamycin; NF-kB, nuclear factor kappa B; PI3K, phosphatidylinositol 3-kinase; PIP2, phosphatidylinositol 4,5-bisphosphate; PIP3, phosphatidylinositol 3,4,5-trisphosphate; PTEN, phosphatase and tensin homolog; S6K, S6 kinase; TAZ, transcriptional coactivator with PDZ-binding motif; YAP, Yes-associated protein.

Through the convergence of biochemical growth factor signaling, mechanical amplification, and duration-dependent stabilization, PI3K–Akt signaling emerges as a major determinant of metabolic identity during cellular proliferation, tissue expansion, and malignant transformation (47) (**Figure 2**).

### 2.5 Mechanotransductive regulation of proliferation in development and tissue repair

The mechanotransductive signaling networks described above also operate under physiological conditions during developmental morphogenesis and tissue repair. In these contexts, convergent mechanical and growth-factor-mediated signals transiently activate pathways such as PI3K–Akt, YAP and TAZ and myocardin-related transcription factor-serum response factor to support controlled proliferative expansion. Embryogenesis represents a highly proliferative phase characterized by a dynamically regulated mechanical environment and coordinated biochemical signaling that shapes morphogenesis, tissue patterning, and organogenesis (58).

During early development, spatial confinement, tissue tension, and extracellular matrix remodeling enhance integrin–focal adhesion kinase signaling, cytoskeletal tension, and nuclear accumulation of YAP and TAZ, alongside the activation of myocardin-related transcription factor-serum response factor transcriptional programs that support rapid proliferative expansion (58, 59). As development progresses, tissue geometry, matrix composition, and mechanical loading evolve, and cellular programs shift from proliferative expansion toward differentiation and maturation as morphogenesis stabilizes.

After birth, similar mechanochemical principles operate during tissue repair. Endothelial disruption, inflammation, and extracellular matrix remodeling generate transient increases in tissue tension, interstitial pressure, and matrix stiffness that support endothelial proliferation, angiogenesis, and fibroblast activation (60, 61). These mechanical cues act in concert with vascular endothelial growth factor, transforming growth factor beta, and related pathways to coordinate wound repair and tissue regeneration (61). As tissue integrity is restored and mechanical tension resolves, YAP and TAZ activity declines through cytoplasmic redistribution, and proliferative signaling diminishes (52, 59).

Wound repair is also associated with metabolic reprogramming toward aerobic glycolysis to support biosynthetic demand, redox balance, and rapid cell division. This shift reflects the PI3K–Akt and HIF-1alpha-dependent metabolic programs described in Section 2.4, here operating transiently in the context of repair. A temporally ordered immune response accompanies this transition, with an initial inflammatory phase initiating repair and a subsequent resolution phase permitting sustained proliferative expansion and tissue remodeling (61, 62).

These observations indicate that biochemical signals alone are often insufficient to sustain prolonged proliferative activity without concurrent mechanical reinforcement. Ligand-driven pathways are frequently attenuated through feedback inhibition, receptor desensitization, and ligand depletion, whereas mechanically derived cues from extracellular matrix remodeling, tissue tension, and microenvironmental restructuring persist until structural homeostasis is restored (58, 61). Developmental and reparative proliferation therefore emerges from coordinated convergence of mechanical activation, growth factor signaling, and metabolic adaptation, including increased glycolytic flux and regulated immune activity (61, 62).

This shared mechanochemical architecture reflects a broader organizing principle. In healthy tissues, proliferative activity is coupled to transient mechanical cues that amplify mitogenic signaling, and declines as these cues diminish. When mechanical stress persists beyond its physiological temporal window, as in fibrosis, chronic inflammation, and sustained extracellular matrix stiffening, integrin–focal adhesion kinase signaling, cytoskeletal tension, and YAP and TAZ activity may remain elevated, reinforcing growth-permissive transcriptional and metabolic programs (59, 63). Persistent mechanotransduction can suppress anoikis, attenuate contact inhibition, and destabilize lineage identity, altering apoptotic thresholds and tissue organization in ways that support a pro-proliferative microenvironment capable of facilitating early tumorigenic progression within mechanically dysregulated tissues (63, 64).

### 2.6 Biophysical dysregulation and tumor initiation: the wound that fails to resolve

Solid tumors share structural and functional similarities with non-resolving wounds, particularly regarding aberrant vasculature, persistent vascular permeability, and sustained inflammatory signaling (65, 66). This concept was articulated by Harold Dvorak, who described cancer as a wound that fails to heal in the context of chronic vascular leak and angiogenic activation (65). The present framework extends this analogy beyond vascular permeability to propose that carcinogenesis may arise as a downstream adaptive response to persistent disruption of tissue-level biophysical homeostasis. In this view, tumor initiation is not driven solely by mutation as a primary event but also reflects cumulative selective pressures generated through sustained mechanochemical dysregulation.

The downstream metabolic, transcriptional, and survival outputs of the mechanotransductive signaling axes established in Section 2.4 collectively provide the basis for this selective pressure. The glucose-committed, anoikis-resistant, and growth factor-independent cellular phenotype that emerges from sustained PI3K–Akt–mTOR and YAP–TAZ activation is not incidental to the mechanovascular framework but is precisely its output: a cellular state in which proliferative commitment, metabolic flexibility, and apoptotic resistance are simultaneously reinforced. Cells operating under these persistent mechanochemical constraints are therefore subjected to cumulative selective pressures favoring the stabilization of phenotypes capable of maximizing survival and resource acquisition within a chronically dysregulated tissue context.

Consistent with tissue organization field theory, this framework proposes that sustained abnormalities in tissue mechanics, interstitial transport, and endothelial and electrostatic barrier properties may precede and shape genetic stabilization rather than arising as consequences of somatic mutation (67). Genetic and epigenetic alterations may subsequently emerge to fix phenotypes already selected under persistent mechanical and metabolic constraints (35, 68). Metabolic intermediates generated under high glycolytic flux, including acetyl-CoA, alpha-ketoglutarate, and NAD+/NADH ratios, influence chromatin-modifying enzyme activity, linking sustained metabolic reprogramming to heritable transcriptional changes that consolidate the proliferative phenotype (68).

This framework applies most directly to solid tissues exposed to chronic mechanical stress, fibrosis, inflammation, ischemia-reperfusion injury, or incomplete repair (66). It is not intended to replace mutation-driven models in hematologic malignancies, where tissue-scale mechanical constraints are less prominent. It rather proposes a temporally ordered relationship in which biophysical dysregulation establishes the selective microenvironment, metabolic and signaling adaptations consolidate a pro-proliferative cellular state, and genetic stabilization subsequently fixes these phenotypes into heritable configurations.

In this view, tumorigenesis represents a stabilized endpoint of unresolved mechanochemical dysregulation in which somatic mutations arise within, and are shaped by, a tissue context already substantially reorganized by sustained biophysical stress.

### 2.7 Glycocalyx disruption and the initiation of microenvironmental dysregulation

Host defense against tissue injury and environmental stress is supported by hierarchically organized barrier systems, among which biophysical and electrostatic structures play early and critical roles. The endothelial glycocalyx represents a key interface regulating vascular permeability, molecular exchange, and cellular interactions (69, 70). This dense, negatively charged, glycosaminoglycan-rich layer contributes to the electrostatic exclusion of macromolecules and pathogens, limits access to adhesion receptors, and maintains vascular barrier selectivity.

The structural integrity of the glycocalyx depends on the organized assembly of its principal glycosaminoglycan components, including heparan sulfate, hyaluronic acid, and chondroitin sulfate, anchored to the endothelial surface through transmembrane proteoglycan core proteins such as syndecans and glypicans (69, 70). Heparan sulfate, the most abundant and functionally significant component, contributes substantially to charge density and barrier function. Its degradation may be mediated by heparanase, released from activated mast cells and inflammatory cells under stress and inflammation, and by matrix metalloproteinases that cleave syndecan ectodomains. Hyaluronic acid is degraded by hyaluronidase and reactive oxygen species under oxidative stress, while chondroitin sulfate is subject to proteolytic cleavage by matrix metalloproteinases. Additional shedding occurs through disintegrin metalloproteinases, including ADAM10 and ADAM17 (71). These enzymatic processes are upregulated by inflammatory mediators including tumor necrosis factor-alpha and interleukin-1 beta, generating a feedback loop in which inflammation promotes further glycocalyx degradation. Glycocalyx disruption has been documented across clinical conditions also associated with elevated cancer risk, including sepsis, ischemia-reperfusion injury, diabetes mellitus, hypertension, atherosclerosis, renal disease, and aging-related endothelial dysfunction (71, 72).

Sustained mechanical stress, oxidative damage, and enzymatic mediators of inflammation act in parallel with endothelial dysfunction to impair vascular barrier selectivity and increase permeability (70–72). Although mechanistically distinct, these processes converge on reduction of the electrostatic and steric barrier properties that normally limit interstitial fluid accumulation.

**Figure 3:**
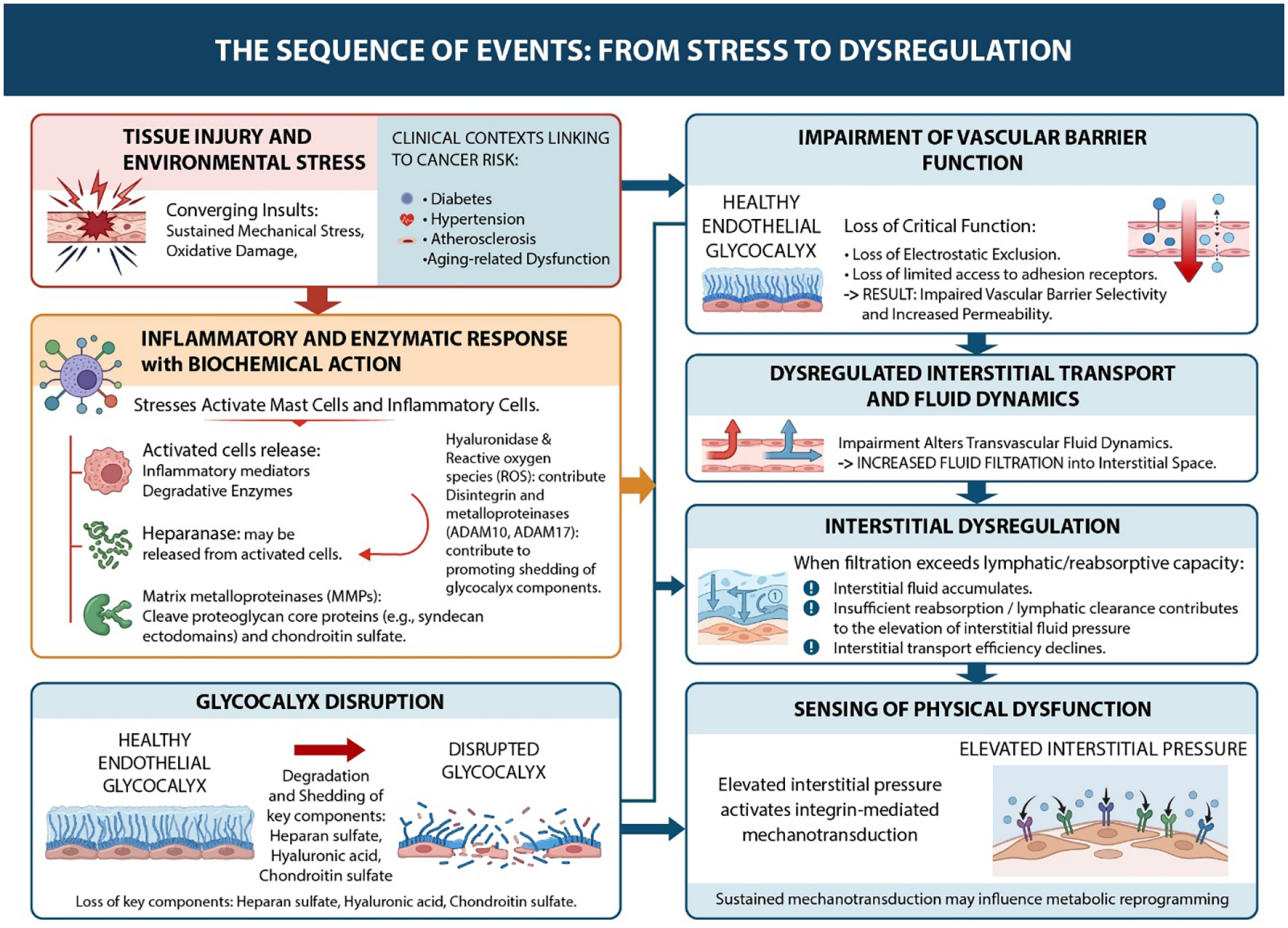
Sequence of events: from stress to dysregulation—glycocalyx disruption and the initiation of microenvironmental dysregulation. A two-column sequential flow diagram illustrating the cascade from tissue injury and environmental stress to stromal mechanosensing. Left column: Tissue injury and environmental stress activate mast cells and inflammatory cells, which release inflammatory mediators, including tumor necrosis factor-alpha (TNF-alpha) and interleukin-1 beta (IL-1 beta), degradative enzymes, heparanase, and matrix metalloproteinases (MMPs) that cleave proteoglycan core proteins, including syndecan ectodomains and chondroitin sulfate. Additional shedding occurs via hyaluronidase, reactive oxygen species (ROS), and disintegrin metalloproteinases (ADAM10 and ADAM17). Clinical conditions linking these insults to elevated cancer risk—including diabetes, hypertension, atherosclerosis, and aging-related endothelial dysfunction—are shown as a sidebar. The resulting glycocalyx disruption involves loss of heparan sulfate, hyaluronic acid, and chondroitin sulfate. Right column: Loss of glycocalyx integrity impairs vascular barrier function through the loss of electrostatic exclusion and reduced access to adhesion receptors, increasing permeability. This alters transvascular fluid dynamics, increasing filtration into the interstitial space. When filtration exceeds lymphatic and reabsorptive capacity, interstitial fluid accumulates, effective lymphatic clearance becomes insufficient, and interstitial transport efficiency declines. The resulting elevation in interstitial pressure activates integrin-mediated mechanotransduction in stromal cells, with sustained mechanotransduction proposed to influence downstream metabolic reprogramming. ADAM10/17, a disintegrin and metalloproteinase 10 and 17; IL-1 beta, interleukin 1 beta; MMPs, matrix metalloproteinases; ROS, reactive oxygen species; TNF-alpha, tumor necrosis factor alpha.

Loss of glycocalyx integrity increases transvascular fluid filtration into the interstitial space. When filtration exceeds lymphatic and reabsorptive capacity, interstitial transport becomes dysregulated. Interstitial fluid accumulates, transport efficiency declines, and interstitial pressure rises. Importantly, this pressure elevation is not solely a passive consequence of fluid imbalance but also acts as a biophysical signal sensed by stromal cells, particularly fibroblasts and endothelial cells, through integrin-mediated mechanotransduction pathways (48, 73, 74) (**Figure 3**).

### 2.8 Matrix expansion, electrostatic filtering, and metabolic partitioning

The mechanotransductive fibroblast activation described in Section 2.7 drives matrix remodeling and a parallel increase in glycolytic flux to provide ATP and biosynthetic intermediates for proteoglycan and glycosaminoglycan synthesis (75). Accumulation of these matrix components increases fixed negative charge density, enhancing Donnan-related osmotic effects and promoting tissue hydration (76). Under physiological conditions, this response is adaptive, allowing transient matrix expansion to buffer mechanical load, limit macromolecular diffusion and pathogen entry, and reduce stress transmission to parenchymal cells.

The quantitative basis for this electrostatic barrier is well established in directly analogous experimental systems. The fixed negative charge density of glycosaminoglycan-rich extracellular matrix in pathologically remodeled interstitium has been experimentally measured at 20 to 100 mEq/liter in cartilage and brain edema models (76), generating a Donnan potential of approximately 5 to 15 mV. For metabolites carrying a net negative charge under physiological pH, including lactate (pKa 3.86, essentially fully ionized at pH 7.4) and pyruvate (pKa 2.49), this electrostatic environment generates a thermodynamic barrier to diffusion that is quantitatively estimable using Nernst–Planck transport equations.

It must be acknowledged, however, that the proposed functional consequences of these electrostatic properties for spatial metabolite heterogeneity in the pre-neoplastic interstitium represent a mechanistic extrapolation that, although grounded in established electrokinetic principles, has not been directly demonstrated for these specific metabolites in human pre-neoplastic tissue. The ionic shielding at physiological ionic strength (approximately 150 mM) produces a Debye screening length of approximately 0.8 nm, which substantially attenuates electrostatic effects at distances beyond a few nanometers. The proposed partitioning effect is therefore most relevant within the dense glycosaminoglycan network of the remodeled pericellular matrix, where molecular diffusivity is simultaneously reduced by steric obstruction and electrostatic repulsion acts over distances comparable to the glycan mesh spacing. Direct experimental validation in pre-neoplastic human tissue is required before causal claims can be made.

When this response persists, continued glycosaminoglycan synthesis progressively elevates fixed charge density and associated osmotic pressure, driving sustained matrix hydration. The extracellular matrix transitions from a compliant, hydration-buffering state to a charge-dense and transport-restrictive state (76, 77). As fixed charge density increases, electrostatic interactions reduce the effective diffusivity of charged solutes, while hydraulic reorganization of interstitial fluid pathways proceeds without necessarily reducing bulk fluid movement.

This produces a functional decoupling of solvent flow and solute transport (77). Water and small neutral molecules continue to move through the hydrated matrix, whereas charged solute mobility becomes increasingly constrained. Convective fluid movement may persist, but effective metabolite transport becomes governed by electrostatic interactions and reduced diffusivity rather than bulk flow. Spatial equilibration within the interstitium is therefore impaired, resulting in persistent concentration gradients and localized retention of charged metabolites within charge-dense stromal compartments (77).

Interstitial lactate arises from stromal glycolysis, parenchymal metabolism, and transvascular flux and is maintained under physiological conditions through vascular reabsorption and lymphatic drainage (74). When transvascular filtration exceeds clearance capacity, interstitial transport becomes dysregulated. Elevated interstitial pressure enhances mechanotransductive signaling in stromal cells, increasing glycolytic flux and lactate production (75) and reinforcing matrix expansion and accumulation of negatively charged matrix components (76). As fixed charge density increases, electrostatic constraints reduce effective lactate diffusivity despite ongoing fluid movement (76, 77), generating spatial heterogeneity with retention of stromal-derived lactate within charge-dense regions and accumulation of vascular-derived lactate in perivascular domains when reabsorption is insufficient (74).

Lactate transport is mediated by proton-coupled monocarboxylate transporters, with MCT1 supporting bidirectional flux and MCT4 functioning predominantly in efflux (34, 35). Under elevated extracellular lactate and reduced interstitial clearance, the transmembrane driving force for lactate efflux is diminished across all resident cells, with net flux determined by intracellular lactate levels and local microenvironmental gradients.

Within this heterogeneous environment, a transport asymmetry emerges. Immune cells migrating through the interstitium encounter regions of elevated extracellular lactate where reduced transmembrane gradients limit efflux, causing intracellular accumulation and impairment of the glycolytic flux required for T cell and natural killer cell activation and effector function (11, 78). These functions are highly sensitive to metabolic constraints, so even modest reductions in lactate clearance may significantly impair immune activity.

Parenchymal cells are not intrinsically shielded from electrostatic constraints. Relative preservation of lactate export rather reflects differences in spatial positioning and local clearance pathways. Because stromal fibroblasts are the principal synthesizers of the glycosaminoglycan-rich matrix that generates transport restriction, they are, by necessity, embedded within the highest fixed-charge-density zones they create; their own lactate efflux is therefore maximally impaired. Parenchymal cells, occupying lower-charge pericellular domains and maintaining communication with lymphatic outflow (74), are comparatively less exposed to the electrostatic barrier at the sites of highest charge density. Reduced vascular reabsorption may be partially compensated by increased lymphatic flow driven by elevated interstitial pressure. Parenchymal cells therefore maintain more effective lactate clearance relative to stromal and perivascular compartments, sustaining glycolytic flux and proliferation.

As matrix charge density increases further, reduced lactate diffusivity and elevated interstitial lactate concentrations diminish transmembrane gradients, producing a functional plateau in lactate clearance. Stromal fibroblasts may consequently shift toward greater reliance on oxidative phosphorylation, reflecting a transport-limited rather than substrate-limited metabolic transition consistent with the PDH regulatory axis described in Section 2.3. As intracellular lactate accumulates and cytosolic NAD+/NADH balance is altered, constraints on mitochondrial pyruvate oxidation are relieved, permitting increased oxidative metabolism (33, 55).

**Figure 4:**
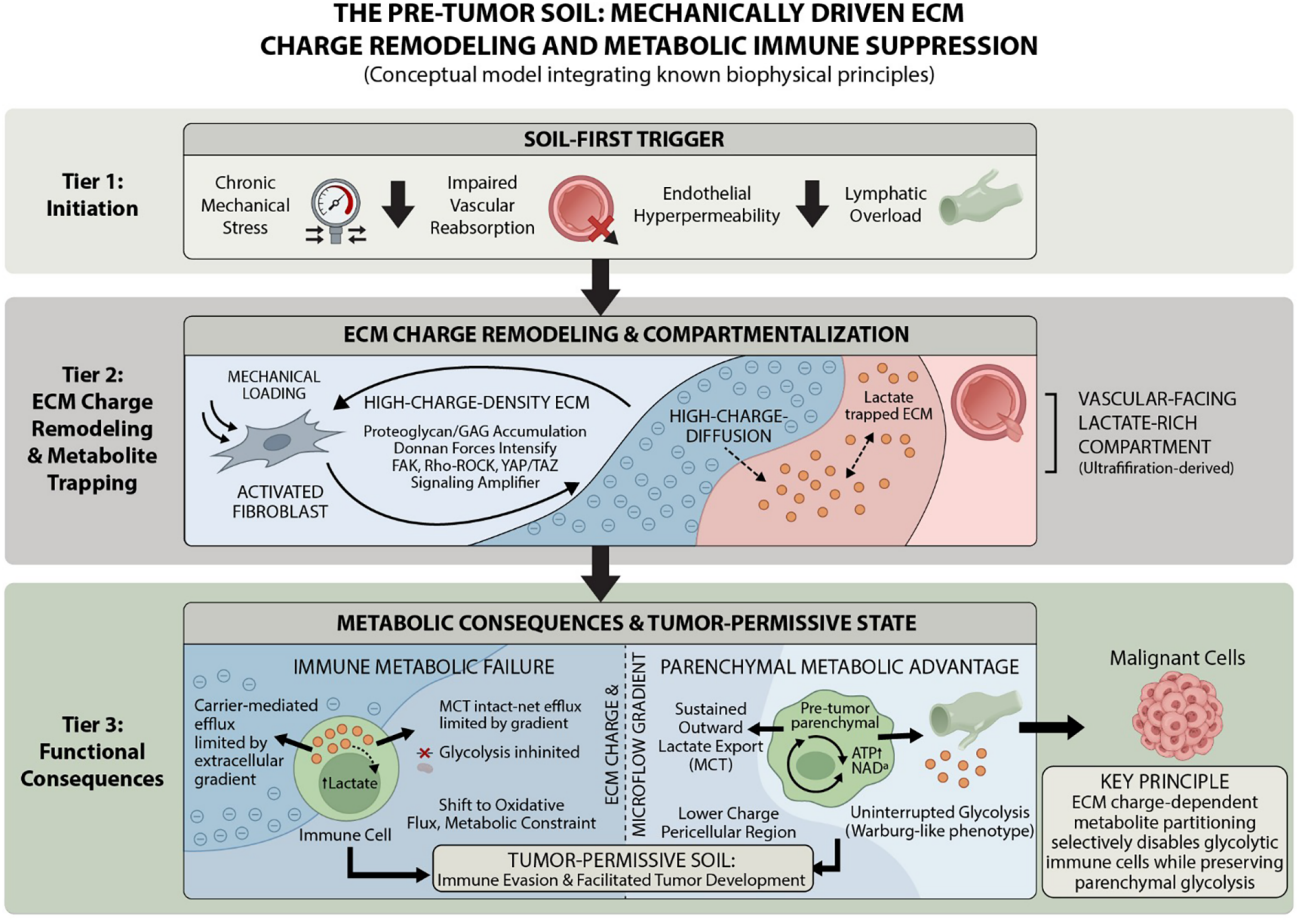
The pre-tumor soil: Mechanically driven ECM charge remodeling and metabolic immune suppression. A three-tier hierarchical flow diagram depicting the progression from biophysical initiating events to spatial metabolic compartmentalization and the emergence of a tumor-permissive microenvironment. Tier 1—Initiation (soil-first trigger): Four converging biophysical insults establish the pre-neoplastic soil: chronic mechanical stress, impaired vascular reabsorption, endothelial hyperpermeability, and lymphatic overload, collectively driving the sustained elevation of interstitial fluid pressure. Tier 2—ECM charge remodeling and metabolite trapping: Elevated interstitial pressure activates stromal fibroblasts, which upregulate glycolytic flux and synthesize proteoglycans and glycosaminoglycans (GAGs), progressively increasing fixed negative charge density. Donnan osmotic forces intensify, and FAK, Rho-ROCK, and YAP/TAZ signaling amplify mechanotransductive output. Lactate accumulation is facilitated by electrostatic and transport constraints within charge-dense stromal regions, while a vascular-facing lactate-rich compartment develops from ultrafiltration-derived accumulation. Tier 3—Metabolic consequences and tumor-permissive state: The interstitium is functionally divided into two metabolic zones. In the immune metabolic failure zone, carrier-mediated lactate efflux in immune cells is limited by the extracellular lactate gradient, MCT-mediated net efflux is constrained by the reduced transmembrane driving force, glycolytic flux is functionally impaired, and intracellular lactate accumulates. In the parenchymal metabolic advantage zone, pre-tumor parenchymal cells in the lower-charge pericellular region maintain sustained outward lactate export via MCT, with preserved ATP generation, NAD+ regeneration, and uninterrupted Warburg-like glycolysis. The key principle is that ECM charge-dependent metabolite partitioning selectively impairs glycolytic immune cells while preserving parenchymal glycolysis, establishing immune evasion and facilitating tumor development. ATP, adenosine triphosphate; ECM, extracellular matrix; FAK, focal adhesion kinase; GAG, glycosaminoglycan; MCT, monocarboxylate transporter; NAD+, nicotinamide adenine dinucleotide (oxidized); Rho-ROCK, Rho-associated coiled-coil-containing protein kinase; TAZ, transcriptional coactivator with PDZ-binding motif; YAP, Yes-associated protein.

These processes establish spatial metabolic partitioning in which electrostatic constraints and transport limitations differentially regulate metabolite distribution. Early impairment of immune cell glycolysis and subsequent stromal metabolic adaptation together suppress immune surveillance while preserving tumor cell metabolism. Persistent matrix expansion thus transitions from an adaptive mechanical response to a charge-mediated transport barrier that reshapes interstitial metabolism and supports a pro-tumorigenic microenvironment, with implications for identifying measurable transport and metabolic markers and for therapeutic strategies targeting interstitial transport dynamics (**Figure 4**).

### 2.9 Convective transport failure and spatial dysregulation of oxygen delivery

Prolonged increases in transvascular fluid filtration elevate interstitial fluid pressure and impose sustained mechanical loading on the interstitium (73). As outlined in the preceding section, these changes are associated with persistent mechanotransductive signaling, extracellular matrix remodeling, and increased fixed charge density, which together may generate electrostatic constraints and alter the hydraulic organization of the tissue, reducing interstitial convection and impairing redistribution of metabolites and protons within the microenvironment.

Impaired oxygen delivery does not appear to arise from intrinsic erythrocyte dysfunction or reduced 2,3-diphosphoglycerate production. Red blood cells retain glycolytic activity within the microcirculation and maintain 2,3-DPG levels via the Rapoport–Luebering shunt, preserving hemoglobin in a release-permissive state (10). This sustained glycolytic activity supports 2,3-DPG production, contributing to reduced hemoglobin-oxygen affinity and facilitating oxygen unloading along the capillary network. Basal oxygen release into plasma therefore remains intact, allowing dissolved oxygen to diffuse into the interstitium according to partial pressure gradients (10).

However, reduced hemoglobin–oxygen affinity alone may not ensure spatially precise oxygen delivery to regions of highest metabolic demand. Under physiological conditions, such spatial regulation depends on the Bohr effect in conjunction with effective interstitial transport (79). Metabolically active cells generate carbon dioxide and protons, which diffuse toward the vascular interface, producing localized pericapillary acidification that promotes oxygen unloading where demand is greatest.

When interstitial transport is impaired, this spatial coupling may become disrupted (74, 79). Although protons and lactate continue to be generated, their redistribution within the interstitium is likely less coordinated, diminishing the spatial precision of pericapillary pH gradients despite ongoing metabolic activity. We propose that this impairment arises not from failure of the Bohr effect itself but from attenuation of the interstitial proton and CO_2_ gradients that normally guide demand-matched oxygen unloading. In parallel, metabolic reprogramming toward aerobic glycolysis generates lactate and protons as primary acidic end products rather than CO_2_, reducing mitochondrial carbon dioxide production per unit glucose metabolized (15). Under these conditions, the Bohr effect relies more heavily on locally generated metabolic acidity; however, if these acidic metabolites are not effectively redistributed through the interstitium, the spatial specificity of this signaling is further compromised.

Consequently, hemoglobin retains its intrinsic capacity for oxygen release, but the localized biochemical cues required for demand-matched unloading are attenuated. Oxygen availability remains permissive, yet its spatial distribution becomes less efficiently aligned with regional metabolic requirements (10, 79), giving rise to functional hypoxia in which overall oxygen delivery capacity is preserved but effective targeting to sites of highest demand is reduced. It is proposed, on the basis of convergent indirect evidence rather than direct experimental demonstration in pre-neoplastic human tissue, that this mismatch arises primarily from dysregulated interstitial transport dynamics rather than from reduced oxygen availability or intrinsic erythrocyte dysfunction. This transport-limited functional hypoxia is mechanistically distinct from classical diffusion-limited hypoxia, which arises from insufficient capillary density in established tumors, and from perfusion-heterogeneity hypoxia arising from structurally abnormal tumor vasculature; the proposed mechanism operates pre-neoplastically with normal capillary density and requires experimental differentiation using spatial pimonidazole staining combined with glycocalyx integrity mapping.

This microenvironmental state may also contribute to immune dysregulation. Local retention of lactate and protons promotes extracellular acidification and metabolically restrictive niches that suppress cytotoxic T cells and natural killer cells (11, 78). Impaired interstitial transport, together with matrix-associated electrostatic constraints, thus contributes to a microenvironment in which oxygen utilization, immune surveillance, and metabolic exchange become progressively uncoupled.

Taken together, the preceding and present sections support a unified model in which extracellular matrix expansion and increased fixed negative charge density contribute to electrostatic filtering and impaired interstitial metabolite transport (76, 77), leading to spatial dysregulation of metabolite and oxygen distribution. Functional hypoxia, commonly attributed to vascular insufficiency or increased oxygen consumption, may arise additionally from a transport-limited state in which impaired redistribution of acidic metabolites reduces the spatial precision of the Bohr effect despite preserved oxygen availability (10, 79). The resulting decoupling of solvent flow and solute transport (77) limits effective clearance and redistribution of lactate and protons, establishing concentration gradients that constrain glycolysis in immune cells while permitting continued lactate export in tumor cells through differential access to clearance pathways (11, 78). These processes together define spatial metabolic partitioning in which lactate accumulation, oxygen delivery, and immune function become progressively uncoupled, promoting immune suppression and supporting tumor progression (73, 74).

### 2.10 Hemodynamic mechanotransduction, vasomotor coordination, and interstitial fluid regulation

As established in Section 1, microvascular fluid balance is governed by the interplay of capillary hydrostatic pressure, plasma oncotic forces, endothelial barrier integrity, and lymphatic drainage (73). Perfusion is therefore regulated not only by cardiac output but also by coordinated local vasomotor control, primarily mediated by endothelin-1 and nitric oxide, with additional modulation from the renin–angiotensin system, metabolic factors such as pH and adenosine, and sympathetic neurohumoral inputs within an integrated hierarchy (80, 81). These regulatory processes operate within a deformable and mechanosensitive vascular network in which mechanical forces and biochemical signaling remain tightly coupled across spatial and temporal scales.

In physiological microvascular beds, vascular behavior cannot be adequately described by simplified applications of Poiseuille’s law. Arterioles and capillaries are compliant, pulsatile, and embedded within dynamically deforming tissues. Flow distribution, pressure transmission, and vascular tone are therefore governed by endothelial mechanotransduction, wall tension, pulsatile hemodynamics, microvascular rheology, and tissue mechanics rather than by purely geometric resistance (82, 83). Vasomotor activity accordingly occurs as a spatially distributed and temporally coordinated process along extended vascular segments rather than as a discrete localized change in resistance.

We propose that vasoconstriction may function as an initiating hemodynamic event that redistributes pressure, shear stress, and flow along the arteriolar–capillary axis in a wave-like manner. To be precise on the hemodynamic mechanism: the elevated capillary hydrostatic pressure proposed here does not arise directly from proximal arteriolar constriction, which in classical resistance analysis reduces downstream capillary pressure. It rather arises from increased outflow resistance generated by mechanical flow impedance within the microvascular network. Such impedance may arise from the physical compression of capillaries by surrounding tissues during cyclic muscular contraction, joint movement, and flexion–extension cycles and is therefore not contingent on vasomotor tone but may occur independently during periods of vasodilation and diastole by the reduced flow velocity. This mechanically imposed outflow resistance elevates capillary hydrostatic pressure transiently during each compressive cycle. When these episodes are recurrent and incompletely dissipated, cumulative elevation of mean capillary pressure may result. Alternatively, pulsatile pressure redistribution imposed by impedance heterogeneity, when proximal constriction increases wave reflection and transmits augmented systolic pressures distally through the compliant capillary network, may contribute to the same end. Under conditions of increased impedance, capillary shear rates may decline, prolonging erythrocyte transit and increasing apparent viscosity (82, 83). The resulting impedance heterogeneity may influence the transmission of subsequent pulsatile pressure waves and generate spatial gradients in mechanical loading along the vascular tree.

These spatially distributed mechanical gradients produce differential endothelial mechanotransductive activation along the vascular axis. The interaction between distal impedance and incoming systolic pressure generates maximal mechanical stimulus in proximal arteriolar segments, with progressive attenuation distally due to viscous energy dissipation. Endothelial cells therefore integrate spatially graded mechanical inputs rather than responding to discrete focal stimuli, enabling coordinated vasomotor responses across extended vascular territories.

When local mechanical loading exceeds a physiological threshold, endothelial cells release endothelin-1 as part of an adaptive vasomotor response, activating adjacent smooth muscle cells to modulate tone (84). This release propagates sequentially along vascular segments with finite temporal delay, producing spatially coordinated regulation of vascular resistance. Under physiological conditions, this mechanotransductive ET-1 response represents adaptive tone regulation that maintains perfusion homeostasis in the face of mechanical perturbation. It is distinct from the pathological ET-1 dominance arising from endothelial injury, which is examined below.

Erythrocytes participate in this vasomotor regulatory loop as dynamic mechanochemical sensors, a role examined in mechanistic detail in Section 2.11. In brief, as red blood cells traverse regions of elevated shear and membrane deformation, glycolytic flux and ATP production are enhanced. When ATP generation transiently exceeds intracellular utilization, a fraction is released into the vascular lumen during capillary transit, with release magnitude scaling with local shear stress and erythrocyte deformation (10, 85, 86). Extracellular ATP activates endothelial purinergic receptors and stimulates nitric oxide synthesis (86, 87), generating a shear-dependent vasodilatory signal that counterbalances endothelin-1-mediated constriction and contributes to restoration of flow distribution. Erythrocytes thus function as flow-responsive modulators of vascular tone, integrating local mechanical conditions into the vasomotor signaling network through shear-dependent ATP release (88).

Endothelial injury or dysfunction disrupts this homeostatic balance by simultaneously amplifying endothelin-1 secretion and reducing nitric oxide bioavailability, whether arising from mechanical stress, inflammation, metabolic imbalance, oxidative stress, or other insults (80, 89). The consequent shift in the vasoconstriction-to-vasodilation ratio elevates arteriolar tone and amplifies pressure transmission toward terminal arterioles and capillaries. Because capillaries possess minimal smooth muscle and limited contractile responsiveness to endothelin-1, the dominant hemodynamic consequence is redistribution and elevation of capillary hydrostatic pressure rather than intrinsic capillary constriction. This injury-associated ET-1 dominance therefore engages the pulsatile transmission mechanism described above: increased arteriolar impedance augments wave reflection and drives sustained systolic pressure transmission distally, elevating mean capillary hydrostatic pressure and directly promoting ultrafiltration when it exceeds effective oncotic pressure (73).

We propose that this ET-1-mediated ultrafiltration initially represents an adaptive hemodynamic response facilitating plasma extravasation, interstitial expansion, and delivery of proteins and signaling molecules required for matrix remodeling and wound repair. The resulting elevation in interstitial fluid pressure imposes mechanical stress on the extracellular matrix and surrounding tissue architecture, activating mechanotransductive signaling in stromal cells and promoting matrix remodeling, increased stiffness, and altered hydration dynamics mediated in part by fixed charge density and Donnan-related osmotic effects (77). In early phases, these processes support tissue repair and structural restoration.

When endothelial dysfunction persists and ET-1-dominant signaling remains unresolved, this adaptive response transitions to a maladaptive state. Sustained elevation of capillary hydrostatic pressure and ultrafiltration drives progressive interstitial fluid accumulation, increased matrix stiffness, and the emergence of electrostatic and hydraulic constraints on interstitial metabolite transport. These interstitial alterations reciprocally modify microvascular hemodynamics by increasing outflow resistance, altering shear distribution, and amplifying impedance heterogeneity in a progressively self-reinforcing manner, coupling vascular and interstitial pathology into a unified mechanovascular disease process.

We propose a unified mechanochemical model in which impedance-mediated and vasomotor pressure redistribution, erythrocyte-mediated ATP–nitric oxide feedback, and interstitial fluid dynamics together regulate microvascular homeostasis. Dysregulation of this integrated system, characterized by persistent ET-1 dominance, impaired shear-dependent vasodilation, and sustained elevation of capillary hydrostatic pressure, may drive chronic interstitial stress, transport limitation, and the emergence of a microenvironment conducive to functional hypoxia, immune suppression, and tumor progression.

### 2.11 Erythrocyte mechanotransduction, shear-dependent glycolytic flux, and ATP-mediated endothelial feedback in microvascular homeostasis

Building on the hemodynamic framework developed in Section 2.10, this section examines the erythrocyte mechanotransductive cascade in greater detail. Red blood cells function not merely as passive oxygen carriers but as active flow-responsive modulators that link shear stress, metabolic kinetics, and endothelial signaling to support tissue perfusion (88). In a deformable vascular network governed by the balance between endothelin-1 and nitric oxide, red blood cells traverse regions of heterogeneous shear, pressure gradients, and transient microvascular impedance, responding dynamically to local mechanical conditions. Unlike proximal elastic vessels, where Windkessel buffering dampens pulsatile energy, the microcirculation operates as a resistance-dominant and mechanically heterogeneous domain in which local shear gradients, impedance, and vasomotor tone influence perfusion distribution and cellular mechanical exposure (90).

Due to the absence of mitochondria, erythrocytes rely exclusively on anaerobic glycolysis for ATP generation, with lactate as an oxygen-independent end product (91). Lactate is exported via monocarboxylate transporters and may subsequently be recycled through gluconeogenesis or utilized by mitochondria-containing tissues such as cardiomyocytes, neurons, renal tubular cells, and oxidative skeletal muscle fibers (92). This metabolic organization allows rapid kinetic adaptation to shear stress, membrane deformation, hydrostatic gradients, and perfusion heterogeneity encountered during microvascular transit. Unlike nucleated cells, erythrocytes regulate metabolic flux primarily through enzyme kinetics, substrate availability, and membrane transport dynamics, enabling rapid biochemical responsiveness without transcriptional reprogramming.

Under conditions of increased endothelin-1-mediated vasomotor tone and elevated distal impedance, erythrocytes may experience increased shear stress and membrane deformation while traversing constricted microvascular segments. These rheological stimuli activate mechanosensitive pathways linked to cytoskeletal strain and ion flux, including channels such as Piezo1, which can enhance glycolytic throughput through existing enzymatic pathways rather than gene regulatory mechanisms (93, 94). Because erythrocyte biosynthetic demands are minimal, this shear-associated increase in substrate flux may elevate ATP generation relative to basal cellular utilization.

Despite increased flux, glycolytic progression remains constrained at key regulatory steps, particularly phosphofructokinase-1 and pyruvate kinase. During high-flux states, these constraints may promote accumulation of upstream intermediates, including 1,3-bisphosphoglycerate, diverting a fraction of glycolytic carbon into the Rapoport–Luebering shunt, which bypasses the phosphoglycerate kinase step, reduces ATP yield per glucose, and increases 2,3-diphosphoglycerate synthesis.

Shear-associated acceleration of glycolysis may therefore produce a high-flux metabolic state characterized by increased ATP production together with elevated 2,3-diphosphoglycerate levels, reflecting flux-driven adaptation rather than maximal energetic efficiency per glucose molecule. Increased 2,3-diphosphoglycerate lowers hemoglobin–oxygen affinity and may facilitate preferential oxygen unloading in mechanically stressed peripheral microvascular regions (95).

Given the low energetic requirements for erythrocyte maintenance, modest increases in ATP generation may transiently exceed intracellular consumption, allowing ATP release through deformation-sensitive membrane pathways during capillary transit as a mechanosensitive intravascular signaling output proportional to shear exposure (86). In parallel, endothelial cells exposed to elevated shear stress release ATP via pannexin and connexin-mediated pathways, acting as an autocrine and paracrine purinergic signal within the microvascular lumen. In contrast to erythrocytes, endothelial ATP turnover remains closely linked to energy-demanding processes such as cytoskeletal remodeling, barrier maintenance, ion transport, and repair under sustained mechanotransductive loading.

Within this coordinated rheological environment, erythrocyte-derived ATP complements endothelial ATP release to form a local purinergic signaling pool. This signal predominantly activates endothelial P2Y2 receptors, and to a lesser extent P2Y1 receptors, stimulating nitric oxide synthesis and localized flow-mediated vasodilation under conditions of elevated resistance and mechanical stress (86). The purinergic ATP–nitric oxide axis thus functions as a shear-dependent compensatory mechanism that moderates endothelin-dominant vasoconstrictive signaling, stabilizes microvascular tone, and supports adaptive perfusion matching within mechanically heterogeneous microvascular networks.

## 3 Metabolic partitioning, lactate retention, and pre-neoplastic immune metabolic suppression

Extracellular lactate accumulation within tissues is commonly attributed to parenchymal glycolysis; however, a substantial fraction may originate from metabolically reprogrammed stromal cells (96). Fibroblasts, pericytes, and related mesenchymal populations exposed to sustained deformation, elevated interstitial pressure, and matrix stiffening undergo mechanotransductive reprogramming characterized by increased glycolytic flux and anabolic activity (97), supporting continuous lactate export while providing ATP and biosynthetic intermediates required for extracellular matrix synthesis and remodeling (98).

This stromal metabolic state can be interpreted within a physiological framework in which transient immune modulation facilitates wound healing and tissue repair. Tissue injury induces endothelial hyperpermeability and alters vasomotor tone, increasing capillary ultrafiltration relative to venular reabsorption and lymphatic drainage. The resulting elevation in interstitial fluid pressure imposes mechanical loading across the interstitial compartment, with stromal and perivascular cells acting as primary recipients of these forces, while progressive stromal stiffening and matrix remodeling modify the local mechanical environment and modulate mechanotransductive signal transmission to parenchymal cells.

During physiological repair, these processes are self-limited and associated with transient immune modulation permitting tissue regeneration while limiting excessive inflammatory damage. When vascular dysfunction and mechanical loading persist, however, extracellular matrix remodeling becomes stabilized beyond the range of physiological adaptation (75). Increased glycolytic activity supports proteoglycan and glycosaminoglycan biosynthesis, generating fixed negative charges that contribute to Donnan osmotic and electrostatic effects (99). Donnan-driven swelling becomes mechanically constrained, converting osmotic forces into internal solid stress, reducing interstitial hydraulic conductivity, increasing hydraulic resistance, and shifting transport toward diffusion-dominated regimes.

As remodeling progresses, the extracellular matrix adopts characteristics of a charge-dense polyelectrolyte network. Such matrices function as electrostatic bandpass filters in which charged particle mobility is reduced through interaction with localized charge domains while uncharged species diffuse more freely (77), increasing tortuosity and imposing interaction-mediated transport limitations for both anionic and cationic solutes.

As established in Section 2.8, interstitial lactate under physiological conditions is cleared through diffusion, venular reabsorption, and lymphatic drainage. When ultrafiltration exceeds clearance capacity, interstitial pressure rises, lymphatic flow increases, and diffusion becomes progressively constrained by matrix remodeling and electrostatic interactions (73, 100). Elevated interstitial pressure enhances lymphatic flow primarily through increased transcapillary and interstitial-to-lymphatic pressure gradients, promoting convective fluid entry into initial lymphatics, with downstream propulsion supported by intrinsic lymphatic contractility and valvular function (6). Lymphatic dysfunction also impairs the egress of antigen-presenting dendritic cells and reduces adaptive immune priming against early transformed cells, contributing to immune ignorance of pre-neoplastic lesions; VEGF-C/VEGFR3-dependent lymphangiogenesis may be paradoxically impaired by chronic interstitial hypertension through heparan sulfate proteoglycan-mediated sequestration of VEGF-C within the remodeled extracellular matrix. This creates a self-reinforcing bidirectional cycle: elevated interstitial fluid pressure impairs lymphatic drainage, while impaired lymphatic drainage further elevates interstitial fluid pressure, progressively amplifying both the hydraulic and immunological consequences of the initial mechanovascular dysregulation.

Within this altered regime, lactate handling becomes spatially compartmentalized. Increased matrix charge density and elevated interstitial pressure impose electrostatic and hydraulic constraints that selectively impair diffusion and convective delivery toward venular structures, limiting parenchymal-derived lactate access to reabsorptive channels and redirecting clearance toward lymphatic pathways. Stromal-derived lactate remains preferentially retained within localized charge-dense microdomains, where reduced molecular mobility restricts access to both venular and lymphatic clearance routes. These transport asymmetries generate a spatially heterogeneous lactate landscape in which perivascular accumulation, efficient lymphatic clearance of parenchymal lactate, and localized stromal retention coexist, transitioning the interstitium from a well-mixed environment to a kinetically compartmentalized system characterized by incomplete equilibration and persistent local gradients (74, 77).

This compartmentalized lactate distribution directly constrains immune cell metabolism. As the extracellular lactate gradient diminishes, intracellular accumulation imposes product inhibition and disrupts lactate dehydrogenase-mediated NAD+ regeneration, limiting glycolytic flux and anabolic metabolism in CD8+ T cells and natural killer cells, which depend on sustained high-rate glycolysis for activation (11, 101).

Immune cells exiting the interstitium through lymphatic drainage are relieved of these localized metabolic constraints upon re-entry into the circulation, where physicochemical conditions permit restoration of normal metabolic flux. Immune suppression in this context therefore arises primarily from microenvironmental transport and metabolic limitations, rather than intrinsic or irreversible immune cell defects, and may remain reversible upon normalization of interstitial physicochemical conditions.

Sustained mechanotransductive signaling and elevated glycolytic activity also influence epigenetic regulation through altered metabolite availability. Lactate accumulation contributes to histone lactylation, increased acetyl-CoA supports histone acetylation, and S-adenosylmethionine availability governs DNA and histone methylation, linking metabolic state to chromatin regulation (68, 102). With prolonged exposure, these adaptations stabilize glycolytic and anabolic phenotypes, promoting transition from transient wound-associated remodeling to persistent pathological states. These epigenetic changes represent a major therapeutic challenge, reflecting cumulative microenvironmental conditioning rather than chronological aging. Carcinogenesis may therefore be conceptualized as the progressive stabilization of a chronically unresolved, mechanically conditioned, and electrostatically regulated microenvironment that suppresses immune surveillance and permits the survival and selection of aberrant cells.

### 3.1 Baseline interstitial pressure and tissue compliance as determinants of mechanosensitivity

Tissue susceptibility to microenvironment-driven pathological remodeling is strongly influenced by its baseline interstitial fluid pressure, which is better understood as an actively regulated physiological state rather than a purely passive hydraulic variable. Under physiological conditions, interstitial fluid pressure in most tissues is maintained within a narrow range, typically slightly subatmospheric to mildly positive, reflecting a dynamic equilibrium among capillary filtration, plasma oncotic forces, endothelial barrier integrity, lymphatic drainage, vascular reabsorption, and prevailing vasomotor tone that collectively determine pre-capillary hydrostatic pressure and interstitial compliance (103). This equilibrium is further modulated by tissue-specific biological inputs, including hormonal signaling, metabolic activity, intrinsic contractility, extracellular matrix composition, and patterns of deformation, establishing relatively stable interstitial pressure ranges that shape tissue-level mechanosensitivity and cellular responsiveness to sustained biochemical and physical cues.

From a biophysical perspective, tissues can be conceptualized along a continuum ranging from relatively low-prestress, highly compliant interstitial environments to comparatively more conditioned and prestressed matrices. Many soft, exchange-oriented tissues such as skin, lung, intestinal mucosa, breast, neural tissue, and hormonally responsive glands generally operate within lower effective interstitial pressure and higher compliance regimes owing to deformable extracellular matrix structure, active lymphatic drainage, and efficient interstitial fluid dissipation (103). In contrast, organs such as the myocardium, skeletal muscle, liver, and certain renal compartments tend to function within more prestressed interstitial environments characterized by denser stromal architecture, cyclic loading, and comparatively lower compliance (104). These distinctions reflect dominant physiological tendencies shaped by tissue architecture, vascular organization, contractile activity, and functional demand rather than absolute categories.

In tissues with relatively low baseline interstitial fluid pressure and compliant extracellular matrix, the matrix is less prestressed and more deformable, allowing forces to be more readily transmitted to stromal and parenchymal cells. Stromal biosynthetic activity and matrix turnover remain dynamically regulated, but structural compliance limits intrinsic buffering capacity. Consequently, even modest but sustained increases in capillary filtration or vasomotor imbalance can produce measurable interstitial expansion and matrix deformation (103). Because intrinsic resistance is limited, pressure-induced strain is more readily conveyed through integrins and cytoskeletal networks, facilitating the activation of mechanotransductive pathways including focal adhesion kinase signaling, Rho-ROCK activation, YAP and TAZ transcriptional regulators, and mechanosensitive ion channels (105). Under such conditions, mechanotransduction may emerge as an early dominant adaptive response to sustained interstitial stress.

Neural tissue represents a specialized example of a compliant, low-prestress interstitial environment in which even small deviations in interstitial pressure can produce significant functional consequences due to limited intrinsic buffering capacity and spatial confinement. Although its extracellular matrix is highly specialized, the neural interstitium operates under relatively low baseline prestress and is highly sensitive to perturbations in pressure and fluid balance, illustrating how compliant interstitial environments may exhibit increased responsiveness to sustained alterations in interstitial pressure, perfusion, or matrix deformation.

Heightened mechanosensitivity in compliant tissues does not inherently imply pathological susceptibility under physiological conditions. Vulnerability emerges primarily when physical inputs become persistent, non-oscillatory, and insufficiently dissipated. When sustained elevations in interstitial pressure, matrix deformation, or vascular dysregulation occur without effective compensation by vascular reabsorption or lymphatic drainage, signals may accumulate and stabilize mechanotransductive activation (106). This effect is further amplified when persistent physical stress coincides with growth-promoting biochemical signaling, including growth factor, hormone-like anabolic signaling, or PI3K–Akt-associated cues, which together lower the threshold for sustained proliferative and metabolic reprogramming.

Physical stimuli must also be distinguished from physiological contractility. In tissues with intrinsic cyclic contractile activity, such as lung and skeletal muscle, rhythmic deformation generates oscillatory interstitial pressure fluctuations that promote lymphatic propulsion and venous reabsorption (107). Lymphatic flow can increase several folds in response to transient elevations in interstitial pressure, providing efficient fluid and solute clearance (73). This oscillatory environment facilitates the dissipation of transient pressure gradients and preserves interstitial homeostasis despite repeated loading, keeping cues dynamic and self-limiting rather than persistently accumulative.

Conversely, tissues or microenvironments exposed to sustained and poorly dissipated physical stress, such as those undergoing chronic inflammation, fibrosis, vascular injury, or unresolved tissue remodeling, may gradually transition toward a mechanically stabilized interstitial state. Stromal cells experience prolonged pressure loading that promotes adaptive increases in collagen, proteoglycan, and glycosaminoglycan synthesis, leading to progressive extracellular matrix reinforcement and altered interstitial transport properties (108, 109). This stromal reinforcement redistributes forces across the matrix and modifies how they are transmitted to parenchymal cells rather than eliminating mechanotransduction.

Parenchymal cells residing in chronically prestressed interstitial environments may undergo adaptive cytoskeletal and membrane remodeling, including increased structural resilience and altered baseline permeability, such that metabolic activation and anabolic signaling become more strongly regulated by mechanically gated pathways operating in concert with chemical gradients. Skeletal muscle provides a physiological example in which baseline glucose uptake is tightly regulated, yet metabolic entry can be markedly enhanced through contraction-induced mechanotransduction in addition to insulin signaling. Exercise-associated loading promotes GLUT4 translocation and glucose uptake even in the relative absence of insulin, illustrating how chronic conditioning elevates the threshold for aberrant mechanosensitive activation while preserving adaptive, stimulus-dependent metabolic flexibility (110, 111).

Although lymphatic drainage and vascular reabsorption generally increase in response to rising interstitial pressure, tissue vulnerability is determined not solely by clearance capacity but also by the temporal pattern of physical inputs (103). Transient or oscillatory stress is typically dissipated effectively, whereas sustained and non-resolving loading can progressively overwhelm clearance mechanisms and stabilize elevated interstitial pressure states, with persistent mechanotransductive signaling, extracellular matrix remodeling, and altered perfusion dynamics becoming self-reinforcing.

Baseline interstitial fluid pressure and tissue compliance thus represent key determinants of the balance between sensitivity, adaptation, and clearance. Relatively compliant, low-pressure tissues prioritize rapid exchange and biological responsiveness, which is physiologically advantageous but may render them more responsive to sustained microenvironmental stress if clearance mechanisms fail. Tissues with relatively prestressed matrices or strong intrinsic contractile activity may exhibit greater resilience through enhanced force dissipation and adaptive remodeling (104). This continuum-based framework links interstitial pressure, extracellular matrix mechanics, mechanotransduction, growth signaling, contractility, and tissue-specific vulnerability, supporting the broader concept that chronic, unresolved physical stress can progressively override physiological regulatory constraints and contribute to the emergence of a proliferation-permissive microenvironment.

### 3.2 The dual axis of tumor evolution: microenvironmental induction and genetic–epigenetic stabilization

Within a hemodynamically compromised niche, fluctuating perfusion together with recurrent hypoxia and reoxygenation cycles promotes reactive oxygen species generation, contributing to DNA damage, replication stress, and progressive genomic instability (112). Under these conditions, cells with TP53 deficiency or impaired checkpoint control acquire a selective survival advantage through reduced susceptibility to apoptosis and the ability to bypass oncogene-induced senescence, which normally constrains aberrant proliferation under stress-associated signaling (113), permitting continued cell cycle progression within a hostile microenvironment and enabling genomically unstable clones to persist and undergo clonal selection (114).

In parallel, progressive extracellular matrix stiffening and elevated interstitial pressure enhance mechanotransductive signaling through the PI3K–Akt pathway and YAP and TAZ transcriptional regulators, reinforcing cytoskeletal remodeling, proliferative signaling, and glycolytic metabolism in early lesions (47, 59). The convergence of metabolic stress, mechanical signaling, and oxidative damage does not directly determine specific oncogenic mutations but instead establishes a permissive selective landscape in which pre-existing or newly arising clones harboring KRAS mutations or MYC amplification gain a competitive advantage (115). These alterations amplify and stabilize microenvironment-conditioned metabolic and proliferative programs rather than serving solely as initiating events, facilitating the transition from transient adaptive responses to sustained oncogenic signaling states (116).

Within this framework, microenvironmental disruption functions as an early and persistent selective pressure that induces a reversible and plastic hyperproliferative state resembling prolonged wound repair physiology (65). Sustained metabolic and physical inputs, including increased glycolytic flux with consequent lactate accumulation, altered acetyl-CoA availability, and redox imbalance, promote progressive epigenetic remodeling, translating chronic environmental signals into semi-stable heritable chromatin states prior to full genetic fixation (102). This epigenetic plasticity allows multiple subclonal populations to coexist transiently, while the constrained microenvironment continuously selects for stress-tolerant clones that retain proliferative capacity (117). Subsequent genetic alterations predominantly act as stabilizing and amplifying events, consolidating these epigenetically reinforced programs into more autonomous growth states (118).

As stress-adapted KRAS- and MYC-driven clones expand within this niche, they actively remodel the microenvironment through processes consistent with niche construction (115). Enhanced glycolysis, increased lactate export, altered angiogenic signaling, and matrix remodeling intensify extracellular acidosis, hypoxia, and tissue stiffness, reinforcing the selective pressures that initially favored their emergence (119). Metabolically aggressive lineages reshape the environment into a more acidic and resource-constrained state that disproportionately disadvantages less adapted clones, functioning as a form of ecological interference competition (120).

Immune dysfunction, arising through the transport-mediated mechanisms detailed in Section 3, emerges early within this microenvironmental phase rather than exclusively as a late consequence of genetic transformation (11). In parallel, sustained cytokine signaling and stress-associated pathways reinforce immune checkpoint activation, including PD-L1 upregulation mediated through interferon-driven JAK-STAT signaling, further contributing to immune evasion within a metabolically constrained niche (121). These processes integrate with the transport-limited lactate retention and compartmentalization described in the preceding section, linking interstitial mechanics, metabolic stress, and immune suppression within a unified framework (122).

These processes emerge within interstitial environments characterized by altered pressure, compliance, and mechanotransductive sensitivity, as described in Section 3.1. These observations support a dual-axis model of tumor evolution in which microenvironmental instability drives the induction of adaptive metabolic and proliferative plasticity, epigenetic remodeling progressively stabilizes these states, and subsequent genetic alterations act primarily as reinforcing events consolidating these adaptations into persistent malignant phenotypes (118). From an evolutionary perspective, tumorigenesis can be understood as a process of selection within a chronically stressed and mechanically conditioned niche coupled with progressive niche construction, resulting in a reinforcing cycle of microenvironmental alteration, immune modulation, and clonal expansion (123). This framework suggests that early correction of microenvironmental dysfunction may prevent subsequent genetic and epigenetic stabilization, providing a potential window for therapeutic intervention.

## 4 Discussion

The mechanovascular framework presented here positions chronic vascular and interstitial dysregulation as a principal upstream driver of the metabolic and immunological alterations that precede tumor formation. Rather than treating aerobic glycolysis, immune suppression, and progressive genomic instability as independent hallmarks arising from somatic mutation, this framework proposes a unified biophysical sequence in which each element emerges as a consequence of sustained disruption to normal tissue mechanics and transport physiology. This perspective is consistent with and extends existing tissue organization field theory, the wound-healing analogy for carcinogenesis, and vascular normalization strategies while contributing specific mechanistic hypotheses regarding the roles of glycocalyx degradation, electrostatic metabolite compartmentalization, and erythrocyte mechanotransduction that have not previously been integrated into a single framework. The evidence assembled here supports the view that interventions capable of restoring vascular–interstitial homeostasis may reduce the likelihood of malignant progression by removing the selective microenvironmental conditions that favor glycolytic reprogramming, immune metabolic suppression, and genomic instability.

### 4.1 Endothelin–nitric oxide imbalance axis

Within this framework, two upstream mechanisms may independently or convergently sustain the chronic ultrafiltration that initiates microenvironmental dysregulation. First, disruption of the endothelial glycocalyx, by biotic insults such as oncoviral infection or *Helicobacter pylori*-driven chronic inflammation or by abiotic exposures, including ionizing radiation and chemical carcinogens, increases transvascular permeability beyond physiological limits and sustains interstitial fluid accumulation independently of vasomotor changes. Second, persistent ET-1 dominance arising from unresolved local hemodynamic trauma, chronic flow impedance, or, in some tissue contexts, capillary rarefaction consequent on failed angiogenic repair elevates effective capillary hydrostatic pressure through a distinct hemodynamic mechanism. Their convergence on elevated interstitial pressure and mechanotransductive matrix remodeling establishes the pre-neoplastic microenvironment described throughout this framework.

Endothelin-1 contributes to microvascular constriction, fibroblast activation, and extracellular matrix deposition in mechanically stressed tissues, often acting in concert with angiotensin II, transforming growth factor-beta signaling, and inflammatory mediators (124). Enhanced endothelin signaling in tumor-associated environments acts as a primary hemodynamic driver, consistent with the capillary hydrostatic-pressure-dependent mechanisms described in Section 2.10, establishing the mechanical and transport alterations that subsequently give rise to functional hypoxia, oxidative stress, and cytokine-mediated inflammation.

Vasoconstriction can increase red blood cell transit velocity and local shear stress within narrowed vascular segments. However, despite this increase in red-blood-cell-associated shear, disturbed flow patterns, endothelial dysfunction, and spatial heterogeneity in shear distribution limit the effectiveness of shear-dependent nitric oxide signaling. Although nitric oxide production may be maintained, it becomes insufficient to counterbalance the elevated endothelin-1-mediated vasoconstrictive drive. This results in a state of functional nitric oxide insufficiency, in which vasodilatory signaling is present but unable to restore vascular homeostasis (125).

The resulting imbalance shifts the microvascular network toward persistent vasoconstriction, reduced functional nitric oxide bioavailability, spatially impaired perfusion distribution, and elevated interstitial pressure. These changes reinforce matrix remodeling, amplify mechanotransductive and metabolic stress signals, and further sustain functional hypoxia.

Targeted modulation of endothelin signaling, together with angiotensin receptor blockade and stromal decompression strategies, may relax microvessels and reduce profibrotic signaling. This may limit excessive matrix deposition and improve perfusion efficiency. Partial decompression could restore shear-dependent endothelial signaling, reduce interstitial pressure, and attenuate mechanochemical feedback loops that sustain vasoconstriction and fibrosis (126, 127).

However, effective intervention should also address upstream drivers of elevated endothelin-1 secretion, particularly endothelial injury arising from both biotic and abiotic causes. Biotic contributors include oncogenic viral infections and chronic bacterial infections such as *Helicobacter pylori*, which promote sustained inflammatory and endothelial stress (128). Abiotic factors include chemical exposures, radiation, mechanical trauma, and environmental toxins, all of which can directly impair endothelial integrity and perpetuate vasoconstrictive signaling (129, 130). Addressing these upstream determinants alongside modulation of vascular tone and matrix dynamics is essential for sustained normalization of microvascular function and prevention of recurrent microenvironmental dysfunction. These considerations are particularly relevant in the context of aging, in which progressive decline in endothelial nitric oxide synthase activity, increased oxidative stress, arterial stiffening, and reduced vascular compliance collectively lower the threshold at which biotic and abiotic insults produce sustained endothelin-1 dominance, rendering aged tissues intrinsically more susceptible to the mechanovascular dysregulation proposed as an upstream carcinogenic driver.

### 4.2 Integration with canonical oncogenic pathways, metabolic plasticity, and intratumoral heterogeneity

It is important to acknowledge that the Warburg glycolytic phenotype, while predominant in many rapidly proliferating solid tumors and mechanistically central to the pre-neoplastic conditions addressed here, is neither universal nor metabolically static. Cancer cells exhibit substantial plasticity, transitioning between glycolytic and oxidative states across cell cycle phases, in response to nutrient availability and under therapeutic pressure (131). Many malignancies display coexistence of glycolytic and oxidative phosphorylation-dependent subpopulations, glutamine addiction, fatty acid oxidation, and metabolic symbiosis between spatially distinct tumor regions (132). Oxidative phosphorylation dominance is increasingly associated with advanced progression, metastasis, chemoresistance, and poor clinical outcome rather than with early tumor initiation, as demonstrated in endocrine therapy-resistant metastatic breast cancers (133, 134), triple-negative breast cancer under chemotherapy (135), and circulating tumor cells undergoing metastatic dissemination (136). Metabolic symbiosis between glycolytic and oxidative subpopulations further contributes to chemoresistance (137). Post-translational regulatory mechanisms, including ubiquitin-mediated proteasomal control of chromatin-associated proteins and oncogenic substrates, represent an additional layer through which the metabolic state of the mechanovascular niche may be transduced into heritable epigenetic changes with direct relevance to colorectal and other solid tumor types arising in chronically stressed tissues (138). The present framework therefore identifies pre-neoplastic glycolytic reprogramming as the initial mechanovascular adaptation while accommodating the full metabolic diversity of established cancers.

The immune-metabolic suppression proposed in this framework involves several distinct mechanistic pathways that require explicit elaboration. Elevated extracellular lactate inhibits NFAT nuclear translocation in T cells through pH-dependent intracellular acidification and direct metabolic constraints on calcineurin activity, suppressing the production of effector cytokines, including IFN-gamma and TNF-alpha, and contributing to a functionally exhausted T cell phenotype prior to engagement of canonical checkpoint pathways. Extracellular acidification from co-exported protons impairs natural killer cell cytotoxicity through pH-sensitive perforin membrane insertion, reducing cytotoxic execution without depleting natural killer cell populations. Adenosine generated by CD39 and CD73 ectonucleotidases in the hypoxic interstitium further suppresses T cell and natural killer cell effector function through A2A receptor-cAMP-PKA signaling, and stromal lactate drives macrophage polarization toward an immunosuppressive M2-like phenotype through GPR81 signaling and histone lactylation of anti-inflammatory gene promoters (139).

The mechanovascular alterations described in this framework converge on precisely those downstream effectors activated by canonical oncogenic drivers. Mechanotransductively sustained PI3K–Akt signaling phenocopies the metabolic outputs of KRAS and PIK3CA mutations, transport-limited functional hypoxia stabilizes HIF-1alpha through the same prolyl hydroxylase inhibition engaged by oncogenic pseudohypoxia, and YAP–TAZ nuclear accumulation under sustained matrix stiffening replicates transcriptional programs that MYC directly activates (140). This convergence suggests that the mechanovascular microenvironment lowers the mutational threshold required for oncogenic pathway autonomy rather than replacing mutational events. Importantly, the causal relationship is not unidirectional: oncogenic glycolysis itself generates lactate accumulation and matrix metalloproteinase activation that amplify the very mechanovascular dysregulation proposed here as upstream, establishing a bidirectional amplification loop in which microenvironmental disruption is both a cause and consequence of early neoplastic transformation. Two temporally distinct phases can therefore be distinguished: an initiation phase, in which chronic mechanovascular dysregulation in histologically normal tissue creates the permissive microenvironmental conditions that lower the threshold for oncogenic transformation, operating before any neoplastic event has occurred, and an amplification phase, in which early transformed cells reciprocally worsen the mechanovascular microenvironment through lactate-driven matrix metalloproteinase activation, HIF-1alpha-driven glycocalyx biosynthetic dysregulation, and VEGF-mediated capillary permeability increases.

The spatial mosaic of mechanovascular stress intensity inherent to this framework predicts a corresponding spatial heterogeneity in metabolic phenotype: regions of greatest glycocalyx degradation and highest interstitial fluid pressure are predicted to exhibit the strongest glycolytic bias, while regions with relatively preserved perfusion may support oxidative metabolism or glutamine-dependent tricarboxylic acid cycle activity. This spatial correspondence between mechanovascular and metabolic heterogeneity constitutes a directly testable prediction. Moreover, sustained YAP–TAZ activation under matrix stiffening may generate cancer-stem-cell-like progenitor populations from parenchymal precursors through direct activation of SOX2, OCT4, and c-Myc expression, providing a mechanovascular basis for cancer stem cell emergence within the pre-neoplastic niche (141).

### 4.3 Limitations and testable predictions

This framework is conceptual and integrates vascular, mechanical, metabolic, and immune processes. While individual components are supported by the cited evidence, their causal sequence in tumor initiation requires direct *in vivo* validation. Most mechanistic evidence is derived from preclinical models, and the specific temporal ordering proposed, from glycocalyx disruption through electrostatic immune suppression to clonal selection, has not yet been validated as a complete sequence in any tumor type. The electrostatic compartmentalization model has not been directly demonstrated in human tumor tissue, and the erythrocyte contribution to interstitial lactate pools remains theoretically extrapolated from cardiovascular physiology. The model complements rather than replaces mutation-driven mechanisms and may be context dependent across tissues.

This framework generates several testable predictions. Elevated interstitial pressure and impaired transport are expected to precede sustained metabolic reprogramming in pre-neoplastic lesions (106). Interventions that normalize vascular tone or reduce mechanical stress should attenuate glycolytic flux and lactate accumulation. Matrix structural and electrostatic properties are predicted to influence the spatial distribution of lactate and other charged metabolites, contributing to compartmentalized metabolic environments (76, 77). Immune dysfunction is expected to correlate with extracellular lactate levels and improve upon restoration of transport and clearance, particularly through reduction of lactate retention within the interstitium (11). Experimental validation should include manipulation of extracellular matrix stiffness or charge density using enzymatic and genetic approaches, modulation of interstitial pressure by lymphatic restoration including VEGF-C/VEGFR3-targeted strategies to overcome heparan sulfate proteoglycan-mediated sequestration of VEGF-C in the remodeled interstitium, metabolic flux analysis in spatially resolved tissue sections, oxygen diffusion imaging, intravital multiphoton microscopy of glycocalyx dynamics, and single-cell spatial metabolomics using MALDI imaging mass spectrometry. A structured summary of testable predictions organized by hypothesis, experimental model, predicted observation, and measurable biomarker is warranted as a validation roadmap (133, 134).

Coordinated correction of vascular, mechanical, and transport dysregulation is expected to produce greater therapeutic benefit than targeting any single axis in isolation (126, 127). Within the present framework, the primary therapeutic objective is not to increase perfusion but to optimize its spatial distribution and reduce the excess capillary hydrostatic pressure that drives pathological ultrafiltration. Two complementary strategies are proposed. First, restoration of glycocalyx integrity would reduce transvascular permeability toward its physiological limit, directly limiting interstitial fluid accumulation without requiring vasomotor intervention. Second, rebalancing the ET-1-to-nitric-oxide ratio through targeted modulation of endothelin signaling or augmentation of shear-dependent nitric oxide production would reduce arteriolar tone and normalize capillary hydrostatic pressure, thereby limiting ultrafiltration at its hemodynamic source. Both strategies are directed at reducing nutrient and fluid delivery to the pre-neoplastic interstitium rather than enhancing it, and are therefore mechanistically distinct from vascular normalization approaches designed for established, hypovascular tumors. The net benefit of these interventions is expected to be greatest at the pre-neoplastic stage, before parenchymal cells have metabolically adapted to the dysregulated microenvironment and before epigenetic stabilization has consolidated the glycolytic phenotype. The engagement of DNA damage response mechanisms, including PARP-1-dependent pathways, within this genotoxically stressed niche further underscores the relevance of targeting upstream microenvironmental drivers rather than individual downstream effectors (142). The potential for ubiquitin-proteasomal regulatory networks to modulate oncogenic substrates within mechanically stressed tissues provides an additional mechanistic layer linking the mechanovascular niche to heritable neoplastic transformation (143). Evolutionary and ecological frameworks for niche-conditioned carcinogenesis further support the concept that microenvironmental selection precedes and shapes genetic stabilization (144). Integrative models of dual-axis tumor progression, in which biophysical and epigenetic axes converge, reinforce the rationale for early microenvironmental interception as a strategy for cancer prevention (145).

### 4.4 Concluding remarks

Tumorigenesis reflects a progressive loss of tissue homeostasis driven by the convergence of mechanical stress, impaired interstitial transport, and metabolic reprogramming. The proposed framework suggests that vasomotor imbalance, glycocalyx loss, endothelial hyperpermeability, and reduced lymphatic clearance arise upstream of genetic change, elevating interstitial pressure and matrix stiffness while favoring sustained glycolytic flux. Transport-limited functional hypoxia emerges from impaired redistribution of acidic metabolites rather than from vascular insufficiency per se, as elaborated in Section 2.9. Interstitial lactate accumulation suppresses immune cell metabolism and reinforces anabolic programs, while mechanotransductive signaling stabilizes these states epigenetically. Genetic alterations such as KRAS and MYC activation likely amplify rather than initiate these processes, and tissue compliance further modulates susceptibility. Viewed through the lens of Paget’s seed and soil hypothesis, the present framework proposes a modified interpretation in which the soil is not a static tissue property but a dynamically dysregulated biophysical environment shaped by chronic vasomotor imbalance, matrix remodeling, and interstitial transport failure. The soil, in this formulation, does not merely receive a transformed seed: it actively prepares one by imposing the mechanochemical and metabolic selection pressures that condition parenchymal cells toward glycolytic commitment, immune evasion, and epigenetic stabilization prior to genetic fixation. These observations together support a model in which microenvironmental disruption precedes genetic evolution, identifying the restoration of vascular and transport function as a primary target for early cancer interception.

## Data Availability

The original contributions presented in the study are included in the article/supplementary material. Further inquiries can be directed to the corresponding author.
