# Correction: A mechanovascular framework for pre-neoplastic microenvironmental dysregulation and early carcinogenesis

**DOI:** 10.3389/fonc.2026.1929886

**Published:** 2026-07-16

**Authors:** Amal Bhanu Vayakkattil, Aiswarya Sivan Pazhanchery, Varsha Vijayarajan, Udayabhanu Vayakkattil

**Affiliations:** 1 Department of Medicine, Al Ameen Hospital Kunnamkulam, Thrissur, Thrissur, India; 2 Department of Medicine, Sapthagiri Institute of Medical Sciences and Research Center, Bengaluru, India; 3 Department of Biochemistry, Gamma High Tech Laboratory, Ollur, Thrissur, India

**Keywords:** aerobic glycolysis, electrostatic metabolite partitioning, functional hypoxia, glycocalyx disruption, interstitial fluid pressure, mechanovascular framework, pre-neoplastic microenvironment

In the published article, the **Abstract** text was inadvertently excluded. This has now been inserted, and reads:

“Aerobic glycolysis is a defining feature of many solid tumors; however, the upstream physiological conditions that initiate and stabilize this metabolic phenotype during early carcinogenesis remain incompletely understood. Here, we propose a mechanovascular framework in which chronic vasomotor dysregulation, endothelial glycocalyx disruption, low-grade inflammation, endothelial hyperpermeability, and impaired lymphatic drainage collectively contribute to elevated interstitial fluid pressure and progressive extracellular matrix remodeling prior to clinically detectable tumor formation. In this context, an endothelin-1-dominant vasomotor imbalance is suggested to increase capillary hydrostatic pressure and promote interstitial fluid accumulation. Erythrocyte mechanotransduction and shear-dependent adenosine triphosphate-nitric oxide signaling are considered integral to microvascular homeostasis, and their disruption may contribute to perfusion heterogeneity and impaired vascular regulation. These biomechanical alterations are associated with the activation of mechanosensitive signaling pathways that enhance glucose uptake and glycolytic flux while constraining mitochondrial pyruvate oxidation, thereby favoring a sustained glycolytic phenotype and cellular proliferation. Progressive matrix expansion increases the fixed negative charge density and may impose electrostatic constraints on solute mobility, contributing to spatial heterogeneity in metabolite distribution. Elevated extracellular lactate levels under these conditions may impair the metabolic fitness of immune cells and reduce their cytotoxic function. We further propose that functional hypoxia may arise from a transport-limited spatial dysregulation of oxygen delivery rather than solely from vascular insufficiency. At the system level, sustained microenvironmental stress is suggested to induce metabolic plasticity, which may be stabilized through epigenetic remodeling and ultimately consolidated by genetic alterations. Collectively, this framework identifies interstitial biomechanical and transport dysregulation as potential upstream drivers of metabolic reprogramming and immune suppression and suggests that restoring vascular-interstitial homeostasis may provide a rational strategy for early cancer interception.”

The original version of this article has been updated.

